# Trial of healthy relationship initiatives for the very early years (THRIVE), evaluating *Enhanced Triple P for Baby* and *Mellow Bumps* additional social and care needs during pregnancy and their infants who are at higher risk of maltreatment: study protocol for a randomised controlled trial

**DOI:** 10.1186/s13063-019-3571-5

**Published:** 2019-08-14

**Authors:** Marion Henderson, Anja Wittkowski, Emma McIntosh, Alex McConnachie, Katie Buston, Philip Wilson, Rachel Calam, Helen Minnis, Lucy Thompson, John O’Dowd, James Law, Elizabeth McGee, Daniel Wight, THRIVE Trial Research Team, Catherine Nixon, Shona Shinwell, Jane White, Karen Crawford, Rosaleen O’Brien, Caoimhe Clarke, Kathleen Boyd, Alice MacLachlan

**Affiliations:** 1 Medical Research Council/Chief Scientist Office Social and Public Health Sciences Unit, University of Glasgow, Top Floor 200 Renfield Street, Glasgow, G2 3AX Scotland; 2 Division of Psychology and Mental Health, School of Health Sciences, The University of Manchester, 2nd Floor Zochonis Building, Brunswick Street, Manchester, M13 9PL England; 3 Health Economics and Health Technology Assessment, University of Glasgow, Glasgow, G12 8QQ Scotland; 4 Robertson Centre for Biostatistics, Boyd Orr Building, University of Glasgow, Glasgow, G12 8QQ Scotland; 5 Centre for Rural Health, University of Aberdeen, The Centre for Health Science, Old Perth Road, Inverness, IV2 3JH Scotland; 6 Institute of Health and Wellbeing, University of Glasgow, Caledonia House, Royal Hospital for Sick Children, Yorkhill, Glasgow, G3 8SJ Scotland; 7 NHS Ayrshire and Arran, Afton House, Ailsa Hospital Campus, Dalmellington Road, Ayr, KA6 6AB Scotland; 8 Institute of Health and Society, School of Education, Communication and Language Sciences, University of Newcastle, Newcastle-upon-Tyne, NE1 7RU England; 9 Parenting and Family Support Research Programme, Department of Psychology and Allied Health Sciences, School of Health and Life Sciences, Glasgow Caledonian University, Cowcaddens Road, Glasgow, G4 0BA Scotland

**Keywords:** Mothers, Parenting interventions, Pregnancy, Maternal mental health, Perinatal mental health, Hard to reach populations

## Abstract

**Background:**

Growing evidence suggests that experiences in the early years play a major role in children’s development in terms of health, wellbeing and educational attainment. The Trial of healthy relationship initiatives for the very early years (THRIVE) aims to evaluate two antenatal group interventions, *Enhanced Triple P for Baby* and *Mellow Bumps*, designed for those with additional health or social care needs in pregnancy. As both interventions aim to improve maternal mental health and parenting skills, we hypothesise that in the longer term, participation may lead to an improvement in children’s life trajectories.

**Methods:**

THRIVE is a three-arm, longitudinal, randomised controlled trial aiming to recruit 500 pregnant women with additional health or social care needs. Participants will be referred by health and social care professionals, predominately midwives. Consenting participants will be block randomised to one of the three arms: *Enhanced Triple P for Baby* plus care as usual, *Mellow Bumps* plus care as usual or care as usual. Groups will commence when participants are between 20 and 34 weeks pregnant.

**Discussion:**

The population we aim to recruit are traditionally referred to as “hard to reach”, therefore we will monitor referrals received from maternity and social care pathways and will be open to innovation to boost referral rates. We will set geographically acceptable group locations for participants, to limit challenges we foresee for group participation and retention. We anticipate the results of the trial will help inform policy and practice in supporting women with additional health and social care needs during antenatal and early postnatal periods. This is currently a high priority for the Scottish and UK Governments.

**Trial registration:**

International Standard Randomised Controlled Trials Number (ISRCTN) Registry, ISRCTN:21656568. Registered on 28 February 2014 (registered retrospectively (by 3 months)).

**Electronic supplementary material:**

The online version of this article (10.1186/s13063-019-3571-5) contains supplementary material, which is available to authorized users.

## Background

Emerging neuroscience evidence suggests that the interactions between a child and their mother, particularly from conception to age 3 years, greatly influence the development of the child’s “base for competence and coping skills that will affect learning, behaviour and health throughout life” [1]. Women with additional health and social care needs in pregnancy (for instance, due to domestic abuse, mental health problems, an addiction, having been in state care, etc.) are likely to be more anxious, depressed and have higher levels of circulating stress-related hormones [2], which can (1) create adverse epigenetic modifications to the developing foetus that permanently affect the baby’s response to stress [3, 4] and (2) independently disrupt the mother’s ability to be sensitive to her baby [5]. Both these pathways may adversely affect the mother-infant interaction. In addition, both poor mother-infant interaction and maternal mental ill health strongly predict child maltreatment [6, 7] and a disadvantaged trajectory for children in terms of their future social, emotional and cognitive development and health [8–10]. Thus, postnatal interventions may not be able to undo all of the changes resulting from maladaptive coping in adverse circumstances during pregnancy.

Sensitive mother-infant interactions are characterised by reciprocal communication from an early stage, which facilitates language but also significantly reduces the risks of maltreatment. This is thought to be due to mothers perceiving their infants as co-operating with them [11]. Mother-infant interactions that succeed in facilitating secure future attachments enable overall development, including language, and may set infants onto a more positive life trajectory. The evidence for the effect of early child development on health and wellbeing in later life is widely accepted and early childhood interventions, such as the High/Scope Perry Preschool Project [12], the USA Family Nurse Partnership (FNP) [13], the Carolina Abecedarian Project [14] and the Chicago Child-Parent Programme [15], have had positive effects on a number of child development domains and on wellbeing and life success in adulthood. Furthermore, early intervention, is generally more cost-effective than intervening later, and is most effective in the antenatal period [16]. However, many applied economic evaluations in the area of home visiting and parenting have had diverse economic objectives and methodological problems including the lack of a societal perspective and limited cost analysis.

We hypothesise that the provision of a parenting support intervention, *Enhanced Triple P for Baby* (ETPB) or *Mellow Bumps* (MB), in addition to routinely provided care during the antenatal and early postnatal phase, will be a cost-effective way of improving maternal mental health, increasing the dyadic synchronicity and sensitivity of mother-infant interactions, reducing the risk of child maltreatment and improving child language and socio-emotional development. The Trial of healthy relationship initiatives for the very early years (THRIVE) is a three-arm, randomised controlled trial of women with additional health and social care needs in pregnancy aiming to compare ETPB plus care-as-usual (CAU) or MB plus CAU with CAU. Based on recent economic evaluation research carried out on a similar trial in Oxfordshire [17, 18], THRIVE will include a comprehensive economic evaluation.

The primary research questions that will be addressed by THRIVE are:

1. Do participants receiving ETPB plus CAU or MB plus CAU have significantly lower anxiety, depression and outwardly directed irritability compared with those receiving CAU alone when their infants are around 6 months old?
2. Do participants who receive ETPB plus CAU or MB plus CAU have more sensitive interactions with their infants compared with those receiving CAU alone when their infants are around 6 months old?

The secondary research questions that will be addressed by THRIVE are:

1. Do infants whose parent(s) receive ETPB plus CAU or MB plus CAU show more cooperative behaviour signs than those whose parent(s) received CAU alone?
2. Do ETPB or MB lead to changes in the number of children flagged as “at risk” on the social services risk register, under a child protection plan, taken into local authority care or attending an accident and emergency department?
3. Do ETPB or MB lead to improvements in the socio-emotional development of children around 30 months old?
4. Do ETPB or MB lead to an improvement in language development in children around 30 months old?
5. Do ETPB or MB lead to an improvement in longer-term educational and health outcomes for children?
6. Is either ETPB or MB cost-effective for the National Health Service (NHS) or society more broadly, in the long term?
7. Do differences in programme fidelity, practitioners’ characteristics and motivation, mothers’ engagement, the intervention mechanisms and contextual factors affect mother and infant outcomes?
8. Does fathers’ involvement or support affect mothers’ engagement with ETPB or MB?
9. How do participants’ experiences of being parented influence their own parenting values and behaviour?

## Methods

THRIVE is a three-arm, randomised controlled trial of 500 participants with additional health and social care needs in pregnancy, classified by National Health Service Greater Glasgow & Clyde (NHS GGC) Special Needs in Pregnancy service (SNiPs) protocol [19] (e.g., domestic violence, mental illness, a history of substance misuse, being looked after in local authority care or criminal justice involvement). Consenting participants will be randomly allocated to one of the three trial arms: ETPB plus CAU, MB plus CAU or CAU alone. All participants and their infants will continue to receive their antenatal and postnatal CAU according to local NHS and local authority guidelines.

THRIVE is sponsored by NHS GGC Health Board (Reference GN12KH589; NHS Greater Glasgow & Clyde, Research and Development Management Office, West Glasgow Ambulatory Care Hospital, Dalnair Street, Glasgow), which is responsible for ensuring that THRIVE is managed “according to the Research Governance Framework for Health and Community Care (Second edition, 2006) and World Medical Association Declaration of Helsinki Ethical Principles for Medical Research Involving Human Subjects”. The study is subject to audit by the NHS GGC Health Board at any time during the trial. In addition, the University of Glasgow Medical Research Council/Chief Scientist Office Social and Public Health Science Unit (MRC/CSO SPHSU) has standard operating procedures that include auditing of ongoing studies.

### Inclusion criteria

Pregnant women aged 16 years and older (or 14 years and older with social work support) who meet the SNiPs criteria, have capacity to consent and are living within the geographical areas of NHS GGC and NHS Ayrshire & Arran (NHS A&A) will be invited to take part in THRIVE when they are between 8 and 24 weeks pregnant. The trial team will use a variety of methods to maximise participant recruitment into the trial:

1. Patients within NHS GGC will be routinely asked if their information can be passed onto researchers. If a pregnant women assents, a THRIVE researcher will contact her to discuss the trial, screen for eligibility and seek consent to recruit to the trial.
2. Participants will be referred by their health or social care practitioner(s) to the research team.
3. Members of the THRIVE team will attend antenatal clinics in hospital and community settings to meet with pregnant women. Participants will be screened by researchers for eligibility.
4. Targeted advertisements, including web-based content, will be used to recruit participants via community centres, health centres, local classified sites, social media and parenting forums. Interested parties will be screened for eligibility by members of the research team.

Following informed, written consent to participate in the trial (Additional file 1), participants will be asked to complete baseline measures after a viable pregnancy has been confirmed through medical records, around the 12th week of pregnancy.

### Exclusion criteria

The following participants will be excluded from the trial:

1. Women with difficulties in speaking or comprehending English, because this can limit their ability to engage in group sessions.
2. Women with no fixed abode, because it may be difficult to trace and retain them within a longitudinal study.
3. Women experiencing active psychosis, because this illness can limit their ability to engage in group sessions.
4. Women for whom it is known during pregnancy that their child will be removed at birth, because doing so may make the separation more difficult.

Advice about the suitability of potential participants to participate in the trial will be sought from the referring health or social care practitioners.

### Interventions

#### Enhanced Triple P for Baby (ETPB)

ETPB, informed by social learning theory, is an intervention designed to facilitate a smooth transition to parenthood for expectant parents by reducing the impact of known risk factors for perinatal distress and difficulties. It aims to provide infants with a healthy start to life by offering parenting skills training as well as psychological coping skills training to parents to enhance parental and infant wellbeing. In addition to focusing on practical parenting and coping skills, group sessions encourage participants to work with their partner or a supportive individual to develop and explore strategies needed to maintain more harmonious family relationships. Each participant’s partner or supportive other is invited to all of the group sessions but participants without support are not excluded. The programme offers four 2-h antenatal group sessions in a community setting and up to three individual 1-h postnatal sessions delivered face-to-face in the woman’s home. The programme is completed with a final 2-h postnatal group session. Antenatal sessions are scheduled to begin before the woman is 34 weeks pregnant, and the postnatal sessions are delivered after their infant is 6 weeks old. ETPB was developed at the Parenting and Family Support Center at the University of Queensland, Australia. Triple P interventions, provided by Triple P International (http://www.triplep.net), are delivered to parents in five different levels of intensity, the efficacy and effectiveness of which have been cited in numerous studies and randomised trials [20, 21]. More recently, however, there has been a call for more registered independent trials (with pre-specified main outcomes), such as THRIVE [22].

#### Mellow Bumps (MB)

MB is underpinned by attachment theory and aims to encourage nurturing and attuned relationships between the mother and developing infant. MB is intended to decrease maternal antenatal stress levels, increase expectant mothers’ understanding of the neonates’ capacity for social interaction and emphasise the importance of early interaction in enhancing brain development and attachment. Participants are encouraged to identify potential sources of stress and work through how to manage these, reflect upon and identify sources of positive social support and to identify barriers to good parenting. The programme offers seven 2-h antenatal group sessions and one 2-h postnatal group session. Antenatal sessions begin when participants are between 20 and 30 weeks pregnant, and the postnatal sessions are delivered when infants are between 6 and 12 weeks old. Partners and/or supportive others are invited to attend one antenatal session. MB was developed by a charity, Mellow Parenting (http://mellowparenting.org), based in Govan, Glasgow. Evidence for Mellow Parenting programmes is small-scale but encouraging [23–25].

#### Care-as-usual (CAU)

All participants in this study will continue to receive their routine antenatal and postnatal care. Generally, this involves care from hospital and community-based healthcare specialists, such as midwives, obstetricians and health visitors. The exact care received by each participant will depend on their individual additional health and social care needs and may also engage services from other sources including social work services and voluntary agencies. All care received will be in line with local authority and Government policy and guidelines [19, 26–30]. The specific services accessed by each participant as part of their CAU will be captured in service use diaries.

Participants randomised to the CAU arm of the trial will not be offered either intervention (ETPB or MB) in THRIVE on completion of data collection. However, participants may access Triple P International and Mellow Parenting programs as part of their CAU, where these are offered through social work or third-sector organisations within NHS GGC and NHS A&A. Uptake of other parenting programmes outside of THRIVE will be captured in the service use diary.

### Trial group organisation

Post randomisation the group sessions will be organised by administrative staff within the trial research team and delivered in community settings, such as local supermarket community rooms or hospital antenatal education rooms. A taxi service will be provided, first, to ensure safe transport of participants to and from the group venue sessions and second, with the aim of reducing anticipated transportation barriers and thereby supporting attendance. The trial administrator will contact participants by phone or text to invite them to sessions, with reminders sent on the day of each group session to maximise group attendance. Attendance at each session will be recorded in an attendance register by group facilitators.

Participation in all aspects of THRIVE will be voluntary, and some participants may choose not to attend any or to attend only some of the group sessions. In these situations, the research team will seek to record the reason for non-attendance. For MB, participants who do not attend either of the first two group sessions will not be invited to subsequent sessions, but will still be eligible for follow-up data collection. For ETPB, participants will be invited to all antenatal group sessions regardless of attendance at previous sessions.

THRIVE group facilitators will be independent of the trial, have a variety of health and social care professional backgrounds, and be employed by NHS GGC and NHS A&A. In order to avoid contamination between intervention arms in the trial group, facilitators will be randomly allocated to deliver only one intervention, the only exception being those with prior knowledge of an intervention - in this instance they will be allocated to deliver that intervention. Training will be conducted by qualified trainers from the host organisation of each intervention.

### Outcome evaluation

Data will be collected at two time points as outlined subsequently.

#### Baseline: 12–24 weeks pregnant

The baseline questionnaire (Additional file 2) will include the following validated measures:

1. The Brief Symptom Inventory (BSI-53; Question (Q)48 (Additional file 2)), which is a 53-item questionnaire covering nine symptom dimensions: somatization, obsession-compulsion, interpersonal sensitivity, depression, anxiety, hostility, phobic anxiety, paranoid ideation and psychoticism [31, 32]. The Global Severity Index from this scale will be used in randomisation to indicate severity of symptoms.
2. The Hospital Anxiety and Depression Scale (HADS), which is a 14-item questionnaire assessing symptoms of anxiety and depression [33]. This will be enhanced by the 4-item outwardly directed irritability (I) questions adopted from the Adult Wellbeing Scale (AWS) [34], as a baseline measure for the primary outcome of maternal mental wellbeing (Q47).
3. The EuroQol 5 Dimensions (EQ-5D-3 L; Q49), which is a standardized instrument to measure health-related quality of life, which will be used for health economic analyses [35].
4. The 23-item Recent Life Events questionnaire (Q45), to determine the experience of specific events in the past year, and whether these affect the respondent [36, 37].
5. The 12-item attitudes and pregnancy to the baby subscale from the Maternal Attitudes and Maternal Adjustment questionnaire (Q32) [38].
6. The 28-item Childhood Trauma Questionnaire (Q89) as a measure of childhood abuse and neglect [39].

The baseline questionnaire will also include questions assessing reasons for vulnerability in pregnancy; socio-demographic characteristics including age, Scottish Index of Multiple Deprivation, ethnicity, education and employment; previous childbirth history and parity; health in pregnancy; and substance abuse [40].

#### Follow up at 6 months postnatal (primary outcome end point)

The primary outcome of maternal mental wellbeing will be assessed by the HADS+I, included in the follow-up questionnaire (Additional file 3; Q35). The primary outcome of mother-infant interaction quality (MIIQ) will be measured based on the CARE Index Dyadic Synchrony Score [41] determined from an episode of play recorded during the follow-up visit when the infant is around 6 months old.

In addition to the HADS+I, the follow-up questionnaire will include the following listed validated measures that will be analysed as secondary outcome measures:

1. The 33-item Support and Control in Childbirth Scale will be used to determine the participants experiences during childbirth (Q14) [42].
2. The EQ-5D-3 L will be used within health economics analyses (Q36) [35].
3. The 23-item Recent Life Events questionnaire (Q41) will be used to determine the experience of specific events in the past year, and whether these affect the respondent [36, 37].
4. The 18-item Cognitive Emotion Regulation questionnaire (Q42) will be used to determine cognitive emotion regulation across nine conceptual scales: self-blame, other-blame, rumination, catastrophizing, positive refocusing, planned, positive reappraisal, putting into perspective and acceptance [43].
5. The 18-item partner subscale of the Postpartum Support Scale (Q68) [44] will be used.
6. The 18-item Parental Regulation Inventory will assess the degree to which parents use specific strategies to encourage or discourage their partner to be involved in parenting (Q69) [45].
7. The 12-item marital relationship scale of the Maternal Adjustment and Maternal Attitudes questionnaire (Q70) will be used [38].
8. The 12-item Multidimensional Scale of Perceived Social Support relating to support from a significant other, family and friends will be used (Q87) [46].
9. The 15-item Karitane Parenting Confidence Scale will be used to assess parenting self-efficacy (Q88) [47].
10. The 14-item Maternal Attitudes Questionnaire will be used to assess attitudes towards motherhood across three domains: expectations of motherhood, expectation of self as a mother and role conflict (Q89) [48].
11. The Parent Coping Scale, a single question will be used to provide a global measure of coping with being a parent (Q92) [49].
12. The 43-item Infant Crying Questionnaire will be used to assess participants’ thoughts/feeling towards infant crying and how they would respond (Q93 and Q94) [50].
13. We will use 16 items from the Postpartum Bonding questionnaire to assess the quality of the mother’s relationship with her baby (Q96) [51].

During the follow-up visit permission will be sought to video-record mother and child interactions around one episode of play and one episode of care, such as feeding or nappy changing. MIIQ will be rated by two research assistants at The University of Manchester (UoM) who will be blinded to the trial arm allocation ensuring single-blind evidence of the effectiveness of the interventions on MIIQ. MIIQ will be measured using both the CARE Index Dyadic Synchrony Score (primary endpoint) [41] and the Mellow Parenting Observation System (MPOS) [52]. As part of this process inter-rater reliability will be established.

As the target population of this study is traditionally seen as “hard to reach”, a number of strategies will be undertaken in order to obtain follow-up data from as many participants as possible, including the offer of £15 vouchers for completed follow-up questionnaires, contact by phone, email and text to arrange follow-up data collection appointments, confirmation of current contact details with the NHS Clinical Research Facility and sending out congratulations cards at birth with a reminder to inform research staff of changes in contact details.

### Economic Evaluation

The economic evaluation will assess the implementation costs, resource use and outcomes associated with each intervention and CAU from the NHS and Personal Social Services (PSS) perspective favoured by the National Institute for Health and Care Excellence (NICE). A broader societal perspective will also be adopted to allow for the possibility of costs and outcomes arising beyond the NHS and PSS, such as housing, education, employment and justice. To facilitate measurement of data on resource use, all participants will be asked to keep a service use diary recording attendance at NHS, social care, local authority and voluntary organisations for both mother and child. The diary will be used as an aide memoir to collect mother and child data between baseline and follow up, and will be used to measure additional and unintentional cost burden/savings associated with the intervention. Participants will additionally be asked to complete a health and social care resource-use questionnaire at follow up based on the same information as the diary.

Up-to-date unit costs will be attached to quantities of resource use to generate mean costs per study participant. The incremental costs and benefits of the treatment arms compared with CAU will be reported within an incremental cost-effectiveness ratio (ICER) if appropriate. The inclusion of the EQ-5D generic outcome measure [35] will also allow the estimation of quality-adjusted life years (QALYs) facilitating cost-utility analysis, the preferred evaluative technique of NICE. Sensitivity analysis will be carried out on the perspective adopted and the key cost-drivers and outcomes. As per recent economic evaluation guidance, missing data will be predicted as a function of relevant baseline covariates [53].

### Process evaluation

Embedded within THRIVE is a realist process evaluation which will carefully monitor what is happening within the trial. Realistic evaluation [54] will provide the theoretical rationale for the process evaluation, which will seek to answer the following questions:

1. How faithfully are ETPB and MB implemented?
2. What are the mechanisms by which they work, if they do, who do they work for and how?
3. What contextual factors are necessary for the programmes to function, or might prevent them from functioning?

The mixed-methods design will include:

1. Pre-training and post-training/intervention questionnaires for practitioners and mothers to be and post-session evaluation forms.
2. Participant observation of practitioner training and the delivery of a series of antenatal sessions.
3. Semi-structured interviews with practitioners, mothers to be, partners and referring practitioners.
4. Telephone interviews with mothers examining the content of the postnatal components of ETPB and MB.

The findings will aid interpretation of the outcomes of THRIVE by providing a greater understanding of how the interventions work (if they do), the extent and quality of their implementation, contextual factors facilitating and constraining intervention functioning, variations in response within and between subgroups of vulnerable parents and benefits or unintended consequences of either intervention. For further information, please consult [55].

**Fig. 1:**
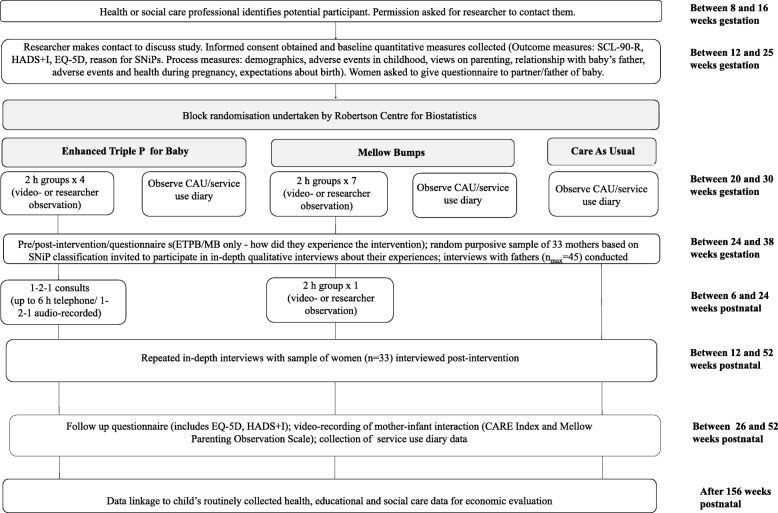
Timeline of participant activities. HADS+1, Hospital Anxiety and Depression Scale enhanced by the outwardly-directed irritability questions adopted from the Adult Wellbeing Scale; EQ-5D, EuroQol 5 Dimensions - a standardised instrument used as a generic measure of health; SNiPs, Special needs in pregnancy; CAU, care as usual; ETPB, Enhanced Triple P for Baby; MB, Mellow Bumps

**Fig. 2:**
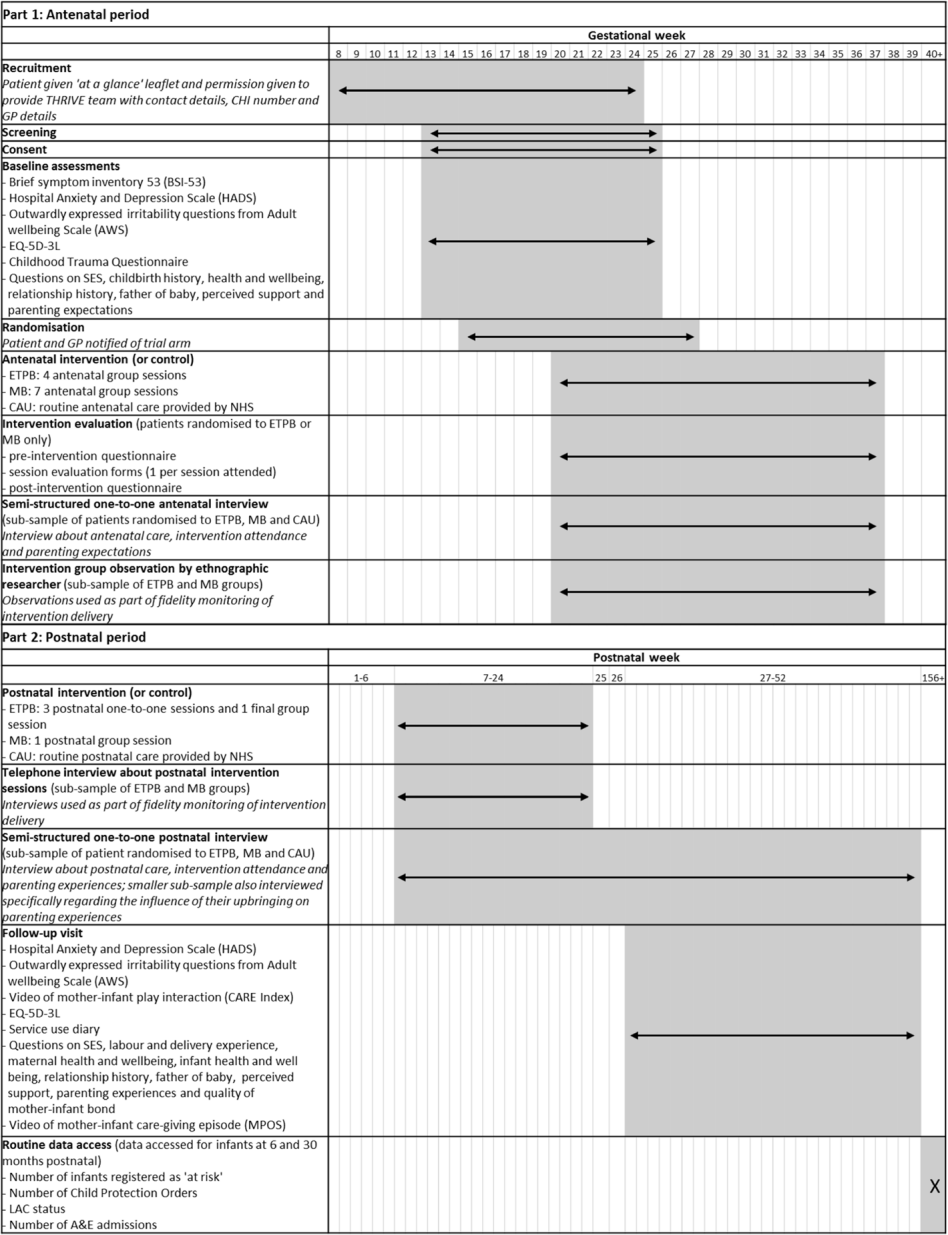
Standard protocol items: recommendation for interventional trials (SPIRIT) diagram indicating the schedule of THRIVE study activities. GP, General Practitioner; EQ-5D-3L, EuroQol 5 Dimensions, 3 Levels - a standardised instrument used as a generic measure of health; CAU, care as usual; ETPB, Enhanced Triple P for Baby; MB, Mellow Bumps; NHS, National Health Service; A&E, Accident and Emergency

A timeline of activities for participants in each arm of the trial is shown in Fig. 1 and a schedule of study activities is shown in a SPIRIT diagram (Fig. 2). A Standard protocol items: recommendation for interventional trials (SPIRIT) checklist indicating where standard protocol items for randomised controlled trials are located within this protocol is included in Additional file 4.

### Planned longer-term follow up using routine data (still to be funded)

Longer-term outcomes will be assessed by linking the short-term outcomes identified in the study to longer-term impacts on health and wellbeing for both mother and child via factors identified from the literature. This trial aspect will rely on linking published associative data and any short-term primary outcomes identified in the trial with long-term costs and benefits in health and education, and other effects such as the effect on employment. Routine data, such as the Strengths and Difficulties Questionnaire, [56] collected by health and social care professionals when the child is around 30 months old, will facilitate the measurement of longer-term outcomes including social and emotional wellbeing, language development, educational attainment, incidences of maltreatment or neglect and placement of children onto the “risk register”/into child protection services. Future linkage will also assess the costs of health, social care and broader education and employment impacts. In addition, it will provide important information in terms of health status and the use of services across the study groups.

All data linkage will be initiated by the Robertson Centre for Biostatistics, which is a registered Clinical Trials Unit (CTU), using the NHS GGC’s Safe Haven that has been developed to support secondary research use of clinical data. Data from NHS A&A will also be required and access to these data via Safe Haven should become available during the life of THRIVE. If this does not occur, then the research team will seek permission from the NHS Information Services Division (ISD Scotland) to link NHS records for NHS A&A’s patients into the Safe Haven.

The research team understands that data linkage at the individual level is sensitive and raises issues of privacy, and will ensure that all data linkage undertaken adheres to the standards outlined by ISD Scotland [57]. In addition, the NHS GGC’s Safe Haven is subject to a local privacy advisory committee that will ensure that that risks to privacy at the individual patient level are minimised.

### Randomisation

Participants will be allocated a unique identification (ID) number by the research team on entry to the study. This ID number will be used to anonymise completed baseline questionnaires, which will be delivered to the CTU for data entry. The CTU will block-randomise participants to the three trial arms using a computer-generated sequence, stratified by parity (number of children), severity of psychiatric symptoms (measured by the BSI, 53) and history of substance dependency, which will be determined based on baseline questionnaire responses. For every 12 participants randomised, two will receive CAU, five will be offered ETPB and five will be offered MB. The research team will receive details of each participant’s intervention arm allocation by email from the CTU based on their ID number. The research team will then contact the participant to let them know which trial arm they have been allocated and to provide details of the group session for those allocated to ETPB or MB. Where consent is provided, the research team will also send a letter to the participant’s health and social care providers to let them know the intervention arm allocation.

### Blinding

Researchers at the MRC/CSO SPHSU, University of Glasgow (UoG) and at Glasgow Caledonian University (GCU) will not be blinded to trial arm allocation due to their direct contact with participants. CTU statisticians and data processers will only receive anonymised data and will not have direct contact with participants. Researchers at UoM will be blinded to trial arm allocation, providing a single-blind evaluation of the quality of mother-infant interactions.

### Storage and anonymisation of confidential data

All data collected will be stored securely, in accordance with the UoG Best Research Practice Guidelines, and managed in accordance with the Data Protection Act 1998 and the General Data Protection Regulation (from May 2018), in either locked filing cabinets or password-protected databases. Data will be accessible only by members of the UoG research team and their research partners at GCU, UoM and the Universities of Aberdeen and Newcastle. All of the data collected will be kept separate from any individual participant identifiers and secure. Participants will be given their own unique ID number in order to link their data throughout the trial. All quantitative data collected as part of the study will be securely transferred to the Robertson Centre for Biostatistics CTU for data entry and cleaning.

Qualitative interview data and recordings of intervention delivery sessions will be transcribed by an external (security checked) transcription agency, and securely stored by researchers based at the UoG and GCU. Video-recordings of mother-infant interactions will be transferred to the UoM using a secure Internet platform designed and maintained by the Robertson Centre for Biostatistics for observer-rated coding using the CARE Index and the MPOS. The one exception to this rule is to allow the secure transfer of a small number of anonymous video-recordings to Mellow Parenting for rater quality-control checks.

In all cases data sharing and confidentiality agreements will be line with the UoG regulations. Permission will be sought from participants for archiving purposes during trial consent, and the trial consent form will notify participants that their data and their child’s data will be made available to the funders in anonymised form.

### Statistical analysis

THRIVE has been powered based on the primary outcome measure of maternal mental wellbeing. The efficacy of ETPB and MB will be analysed at a significance level of 2.5% in order to maintain an overall type I error rate of 5%. Two analyses, performed in a hierarchical fashion so that no further *p* value adjustment is necessary, will be undertaken. In analysis 1, the combined intervention groups (ETPB and MB) will be compared with CAU. If analysis 1 detects a difference at a 2.5% level of statistical significance, analysis 2 will compare ETPB with MB as a primary analysis, to be judged at a 2.5% statistical significance level. If analysis 1 does not yield a statistically significant result, then analysis 2 will be considered a secondary analysis.

#### Power calculation

In order to have 90% power to detect an effect size of 0.4 for Analysis 2 (ETPB versus MB) 157 participants are required in both the ETPB plus CAU and MB plus CAU groups. Comparing these 314 participants with CAU alone in analysis 1 (ETPB and MB vs CAU), requires 61 participants in the CAU alone group to achieve 90% power. Therefore, 375 participants are required in a ratio of ETPB:MB:CAU of approximately 5:5:2. To allow for 25% attrition at follow up, 500 participants will be recruited and randomised.

#### Planned analysis

Baseline-adjusted linear regression analysis (analysis of covariance) will be used to compare primary statistical outcomes between intervention groups. Similar methods will be used for other outcomes (using data transformation and alternative regression methods, depending on the distribution of each outcome). Regression models will be extended to investigate the effects of baseline characteristics and the potential moderating effects of these variables and other measures of intervention compliance. Repeated measures methods will be applied to outcomes collected at more than one post-baseline assessment.

For participants discontinuing from the study before the postnatal follow-up data collection, baseline data will continue to be analysed when consent is provided. Missing values will not be imputed. The sensitivity of the primary outcome analysis to missing values will be explored using multiple imputation, generating 10 imputed data sets, using all available data to predict missing values. Analyses will be conducted for the intention-to-treat population, consisting of all participants who have been randomised to the trial. In addition, analyses will also be undertaken for the per-protocol/on-treatment population.

For the economic analysis the incremental costs and benefits in each treatment arm will be reported within an incremental cost-effectiveness ratio (ICER) if appropriate. Within-trial analysis of costs and effects will be undertaken in STATA (StataCorp, TX, USA), adhering to good practice for economic evaluations alongside clinical trials [58, 59]. Missing data will be explored by employing multiple imputation methods using alternative configurations of baseline covariates [53, 60]. Based on the availability of linked routine data, longer-term costs and outcomes will be assessed by linking short-term outcomes identified in the study to potential longer-term impacts on health and wellbeing for both mother and infant, via trial extrapolation methods including economic modelling techniques.

### Monitoring

#### Data Monitoring Committee (DMC)

The DMC, chaired by Professor John Norrie (j.norrie@ed.ac.uk), will meet regularly (as required) to assess trial progress based on independent trial data (e.g., actual recruitment to trial against projected trial recruitment), provided by the Robertson Centre for Biostatistics CTU and will be given the power to stop the trial should the committee see fit.

If analysis of follow-up data shows that maternal psychological distress significantly worsens after participation in either intervention, we shall seek guidance from the Trial Steering Committee (TSC) and DMC about how to respond, as by that time all of the participants allocated to the ETPB and MB arms will have already received an intervention.

#### Trial Steering Committee

THRIVE will be supported by a TSC comprising academic and health professionals, all of whom are specialists in early years and/or maternal mental health. The TSC will also have a lay member. The committee will be chaired by Professor Rudi Dallos, Plymouth University (R.Dallos@plymouth.ac.uk). It will meet once or twice a year (as required) to provide advice to the research team. Exceptional meetings can be called by the Chair or the Chief Investigator (CI) if guidance is sought as a matter of urgency. Publication plans will be discussed in full with the TSC.

#### Potential harms

- *Data collection*

THRIVE will adhere to Good Clinical Practice guidelines on safety reporting in clinical trials. Our quantitative data collection procedure will consist of completing repeat questionnaires with trained researchers (including Good Clinical Practice training), all of whom will have undergone criminal background checks and have significant experience of working with vulnerable groups. Whilst the questionnaire consists of well-established measures that are not known to be problematic, it is possible that participants may become upset whilst completing the questionnaires. All researchers will receive training in how to handle participant distress and will follow NHS guidance relating to patient confidentiality and protection, including vulnerable adult and child protection procedures. A “useful contacts” sheet will be given to all participants to signpost them to relevant service providers.

Should a researcher become concerned about a participant, s/he will raise this concern with relevant health and/or social care professionals to ensure the participant receives the adequate care they require. In this situation, researchers will complete an incident report form outlining the steps taken and a decision will be made by the CI, Project Manager (PM) and Sponsor as to whether the incident should be reported as an adverse event. The same risks are present in conducting qualitative interviews with participants. All interviewers will have experience in conducting qualitative interviews and working with vulnerable populations and will follow the aforementioned procedures if participant distress is evident.

While researchers may signpost participants to further support services, or, with the participant’s consent, contact their health or social care providers to initiate ongoing support, no formal ongoing post-trial support will be offered by the research team themselves.

b) *Participation in the interventions*

We will be working with vulnerable women at a sensitive period in their lives, and some activities, such as being asked to reflect upon past experiences, may have the potential to cause distress: however, other research suggests asking about adverse childhood events does not cause measurable distress [61]. Furthermore, we believe that this risk is minimal because both interventions are designed to reduce stress through positive action and the development of coping strategies. In addition, the group facilitators will undergo training to work with this group of women and will be able to provide empathetic support and signpost participants to services should they require additional support. The group dynamics may help to reduce stress/distress to participants by providing a supportive and considerate atmosphere in which issues can be discussed. However, it must be recognised that the delivery of ETPB and MB within group settings carries a risk that participants might choose to discuss issues raised with others outside of the group setting. As the focus of the group sessions is more on activities and active discussion rather than disclosing personal histories, the risk of this occurring is low. Nevertheless, to promote respect and confidentiality amongst participants the intervention facilitators will work with them to establish group rules about confidentiality, especially in relation to social media. Furthermore, the bringing together of vulnerable participants may result in the formation of positive or negative group interactions and social networks. Group facilitators will be given the adequate support and resources necessary to signpost participants to appropriate services. At all times, groups facilitators will adhere to NHS guidance relating to patient confidentiality and protection, including vulnerable adult and child protection procedures, and report any concerns about participants to both the PM and their line manager at the NHS GGC Clinical Research Facility; the CI will also be kept informed and be able to influence decision-making.

Should a participant have any concerns about their participation in THRIVE they will be advised to contact the trial PM, CI or the University of Glasgow, Institute of Health and Wellbeing Research Support Manager. These contact details will be included in the trial information booklet given to each participant before they consent to participate in the trial.

- *Withdrawal from the interventions or trial*

Participants may choose to withdraw from the intervention group or the trial at any point. Participants will be asked a reason for withdrawal, but will not be required to respond. Participants who withdraw from the intervention but not the trial will still be eligible for follow-up data collection.

Any women that report experiencing harm from attending the group will be given the support discussed previously and advised by the research team to stop attending the groups. Participants may also be advised by their health or social care practitioner to stop attending groups or withdraw from the trial, based on ongoing health or social care needs. Any participant found to be inflicting harm on another participant or group facilitator will be asked to leave the group sessions and will be withdrawn from further data collection.

It will not be appropriate for participants who no longer meet the study eligibility criteria due to intrauterine death or stillbirth to be invited to subsequent group sessions, and no further intervention or follow-up assessment will be offered to these participants. Women who give birth prematurely while the antenatal sessions of each intervention are ongoing will no longer be eligible to attend antenatal elements of the group but will be offered the postnatal sessions.

#### Safety reporting

The research team will ask permission from participants to notify their General Practitioner (GP) and/or other relevant health and social care workers of her participation in the research. All participants will be told during the consent process that if a significant risk of harm to themselves or their baby/child(ren) is identified, the research team will notify their GP and/or other relevant health and social care professional(s). As previously discussed, structures are in place for group practitioners and researchers to follow should incidents occur relating to the safety of participants and/or their children. When there is a health risk or medical emergency, procedures will be followed including alerting emergency services, GPs or social work services as appropriate. Incidents of this nature, or serious adverse events (SAEs), will always be reported to the PM who will inform the CI.

#### Recording and reporting of adverse events

Any SAE (Table 1) occurring to a research participant will be reported to the NHS West of Scotland Research Ethics Committee (REC), which gave a favourable opinion on the study, when in the opinion of the CI the event was:

1. Related - that is it resulted from administration of any research procedures, and
2. Unexpected - that is the type of event is not an expected occurrence.

**Table 1:** Definition of serious adverse events

Any adverse event or adverse reaction that:	
a. Results in death	
b. Is life threatening	
c. Requires hospitalisation or prolongation of existing hospitalisation	
d. Results in persistent or significant disability or incapacity	
e. Consists of a congenital anomaly or birth defect	
f. Is otherwise considered medically significant by the investigator	
g. Is important but is not immediately life threatening or does not result in death or hospitalization, but may jeopardise the subject or may require intervention to prevent one of the other outcomes listed in the definitions above	

Reports of related and unexpected SAEs will be submitted to the REC within 15 days of the CI becoming aware of the event, using the report of SAE form for non-Clinical Trial of an Investigational Medicinal Product (non-CTIMPS) published on the National Research Ethics Service (NRES) website (https://www.hra.nhs.uk/approvals-amendments/managing-your-approval/safety-reporting/).

#### Annual safety reporting

The CI is also responsible for providing an annual progress report to the REC using an NRES ‘Annual Progress Report form for all other research’. This form is available at https://www.hra.nhs.uk/approvals-amendments/managing-your-approval/progress-reports/.

### Dissemination of data and findings

Primary results will be published in an open access peer-reviewed journal after completion of the trial. Results will also be made publicly available via the trial registration (http://www.isrctn.com/ISRCTN21656568) and THRIVE website (http://thrive.sphsu.mrc.ac.uk/). Participants are entitled to a lay copy of a summary of the findings should they wish.

The final anonymised data set will initially be made accessible to researchers and the public after trial completion by request to the CI, with appropriate confidentiality agreements, with the intention to subsequently publish the data in an online open access archive 5 years after study completion.

## Discussion

THRIVE will explore if women with additional health and social care needs and their infants benefit from participating in group-based antenatal and early postnatal interventions plus CAU more than from CAU alone, and whether these interventions are cost-effective. The interventions are ETPB and MB (see “The interventions”). In particular, we aim to investigate if the interventions in addition to CAU can improve maternal mental health and mother-infant relationships relative to receiving routine care alone.

The participants we aim to recruit are traditionally described as a “hard to reach” population, and THRIVE will be one of the largest definitive trials to date in this population in the perinatal period. We will be monitoring referrals by midwives and social care pathways and be open to innovation to increase the number of referrals and their quality (in terms of eligibility for the trial and communication of referral criteria).

We anticipate the results of the trial will add to the literature on perinatal help and help inform policy and practice in supporting women with additional health and social care needs during antenatal and early postnatal periods.

## Trial status

This publication is based on protocol version 3.0, 31 October 2017. Dates for previous versions of the THRIVE protocol are listed in Table 2. The Sponsor and the NHS West of Scotland Research Ethics Committee approved all protocol modifications, and all changes were communicated to relevant parties in a timely manner. Recruitment for the trial was conducted between January 2014 and March 2018.

**Table 2:** Summary of protocol versions and dates

Protocol version	Date
V3.0	31/10/2017
V2.1	22/04/2016
V2.0	29/10/2015
V1.5	03/12/13
V1.4	17/09/13
V1.3	10/07/13
V1.2	Internal working doc.
V1.1	Internal working doc.

### World Health Organisation registration

Details of the World Health Organisation registration data set are shown in Table 3.

**Table 3:** World Health Organisation Trial Registration Data Set

Data category	Information
Primary registry and trial identifying number	ISRCTN: 21656568
Date of registration	28/02/2014
Secondary identifying numbers	
Sources of monetary or material support	National Institute of Health Research (PHR Project: 11/3002/01) Chief Scientist Office and Scottish Government (GN12KH589 THRIVE)
Sponsor	NHS Greater Glasgow and Clyde Health Board
Contact for public queries	AM Alice.MacLachlan@glasgow.ac.uk
Contact for scientific queries	MH Marion.Henderson@glasgow.ac.uk
Public Title	THRIVE: Trial of Healthy Relationship Initiatives for the Very Early years
Scientific Title	Trial of healthy relationship initiatives for the very early years (THRIVE), evaluating Enhanced Triple P for Baby and Mellow Bumps for those with additional social and care needs during pregnancy and their infants who are at higher risk of maltreatment: study protocol for a randomised controlled trial
Countries of recruitment	Scotland
Health conditions or problem studied	Pregnancy with additional health and/or social care needs such as mental ill health, substance abuse, homelessness or domestic violence
Interventions	Mellow Bumps Enhanced Triple P for Baby
Key inclusion criteria	Pregnant, aged 16 and over with an additional health and/or social care need living in the geographical areas of NHS Greater Glasgow and Clyde and NHS Ayrshire and Arran
Key exclusion criteria	1. Difficulties understanding written and verbal English
	2. Active psychosis
	3. Homelessness
	4. Child will be removed at birth
Study type	Longitudinal randomised control trial
Date of first enrolment	30/01/2014
Target sample size	500
Recruitment status	Recruitment complete
Primary outcomes	1) Do participants receiving ETPB plus CAU or MB plus CAU show significantly lower anxiety, depression and outwardly directed irritability compared to those receiving CAU alone when their infants are around 6 months old?
	2) Do participants who receive ETPB plus CAU or MB plus CAU show more sensitive interactions with their infants compared to those receiving CAU alone when their infants are around 6 months old?
Key secondary outcomes	1) Do infants whose parent(s) receive ETPB plus CAU or MB plus CAU show more cooperative behaviour signs than those whose parent(s) received CAU alone?
	2) Do ETPB or MB lead to changes in the number of children flagged as ‘at risk’ on the social services risk register, under a child protection plan, taken into local authority care or attending an accident and emergency department?
	3) Do ETPB or MB lead to an improvement in the socio-emotional development of children at around 30 months old?
	4) Do ETPB or MB lead to an improvement in language development in children at around 30 months?
	5) Do ETPB or MB lead to an improvement in longer term educational and health outcomes for children?
	6) Are either ETPB or MB cost-effective for the National Health Service (NHS) or society more broadly, in the long-term?
	7) Do differences in programme fidelity; practitioners’ characteristics and motivation; mothers’ engagement; the intervention mechanisms; and contextual factors affect mother and infant outcomes?
	8) Does fathers’ involvement or support affect mothers’ engagement with ETPB or MB?
	9) How do participants’ experiences of being parented influence their own parenting values and behaviour?

## Additional files

Additional file 1: Information sheet and consent form (v3.0 06.09.17). Information sheet provided to participants before study participation and consent form to be completed by all enrolled participants. (PDF 454 kb)

Additional file 2: THRIVE baseline questionnaire (v1.5 15.11.13). Questionnaire completed by study participants at the baseline visit (12–24 weeks gestation) (PDF 5336 kb)

Additional file 3: THRIVE follow-up questionnaire (v2.1 25.01.16). Questionnaire completed by study participants at the follow-up visit (6 months postnatal). (PDF 2561 kb)

Additional file 4: Standard protocol items: recommendations for interventional trials (SPIRIT) checklist. SPIRIT checklist indicating the location of relevant information with this publication. (DOC 121 kb)

## Data Availability

Not applicable

## Abbreviations

A&A: Ayrshire and Arran
AWS: Adult Wellbeing Scale
BSI-53: Brief Symptom Inventory - a short version of Symptom Checklist-90-Revised: measures the severity of any psychological distress across nine dimensions
CAU: Care-as-usual
CHI: Community Health Index
CI: Chief Investigator
CSO: Chief Scientist Office
CTU: Clinical Trials Unit
DMC: Data Monitoring Committee
EQ-5D: EuroQol 5 Dimensions - a standardised instrument used as a generic measure of health
ETPB: Enhanced Triple P for Baby
FNP: Family Nurse Partnership
GCU: Glasgow Caledonian University
GGC: Greater Glasgow and Clyde
GP: General Practitioner
HADS+I: Hospital Anxiety and Depression Scale enhanced by the outwardly-directed irritability questions adopted from the Adult Wellbeing Scale
ICER: Incremental cost-effectiveness ratio
ISD-Scotland: Information Services Division - Scotland
LAC: Looked after children
MB: Mellow Bumps
MIIQ: Mother-infant interaction quality
MPOS: Mellow Parenting Observation Scale
MRC: Medical Research Council
NHS: National Health Service
NICE: National Institute for Health and Care Excellence
NIHR-PHR: National Institute for Health Research Public Health Research Programme
Non-CTIMPS: Non-clinical trial of an investigational medicinal product
NRES: National Research Ethics Service
PM: Project Manager
PSS: Personal Social Services
Q: Question
QALY: Quality-adjusted life years
REC: Research Ethics Committee
SAE: Serious adverse event
SCL90-R: Symptom Checklist--90-Revised
SES: Socioeconomic status
SNiPs: Special needs in pregnancy
SPHSU: Social and Public Health Science Unit
THRIVE: Trial of health relationship initiatives for the very early years
TSC: Trial Steering Committee
UoG: University of Glasgow
UoM: University of Manchester
