# Correction to: Trial of healthy relationship initiatives for the very early years (THRIVE), evaluating Enhanced Triple P for Baby and Mellow Bumps for those with additional social and care needs during pregnancy and their infants who are at higher risk of maltreatment: study protocol for a randomised controlled trial

**DOI:** 10.1186/s13063-019-3674-z

**Published:** 2019-09-10

**Authors:** Marion Henderson, Anja Wittkowski, Emma McIntosh, Alex McConnachie, Katie Buston, Philip Wilson, Rachel Calam, Helen Minnis, Lucy Thompson, John O’Dowd, James Law, Elizabeth McGee, Daniel Wight, THRIVE Trial Research Team

**Affiliations:** 1 Medical Research Council/Chief Scientist Office Social and Public Health Sciences Unit, University of Glasgow, Top Floor 200 Renfield Street, Glasgow, G2 3AX Scotland; 2 Division of Psychology and Mental Health, School of Health Sciences, The University of Manchester, 2nd Floor Zochonis Building, Brunswick Street, Manchester, M13 9PL England; 3 Health Economics and Health Technology Assessment, University of Glasgow, Glasgow, G12 8QQ Scotland; 4 Robertson Centre for Biostatistics, Boyd Orr Building, University of Glasgow, Glasgow, G12 8QQ Scotland; 5 Centre for Rural Health, University of Aberdeen, The Centre for Health Science, Old Perth Road, Inverness, IV2 3JH Scotland; 6 Institute of Health and Wellbeing, University of Glasgow, Caledonia House, Royal Hospital for Sick Children, Yorkhill, Glasgow, G3 8SJ Scotland; 7 NHS Ayrshire and Arran, Afton House, Ailsa Hospital Campus, Dalmellington Road, Ayr, KA6 6AB Scotland; 8 Institute of Health and Society, School of Education, Communication and Language Sciences, University of Newcastle, Newcastle-upon-Tyne, NE1 7RU England; 9 Parenting and Family Support Research Programme, Department of Psychology and Allied Health Sciences, School of Health and Life Sciences, Glasgow Caledonian University, Cowcaddens Road, Glasgow, G4 0BA Scotland

**Correction to: Trials (2019) 20:499**

**https://doi.org/10.1186/s13063-019-3571-5**

Following publication of the original article [1], it has been brought to our attention that an error was slipped into the article’s title.

- Initially published title:
  Trial of healthy relationship initiatives for the very early years (THRIVE), evaluating Enhanced Triple P for Baby and Mellow Bumps additional social and care needs during pregnancy and their infants who are at higher risk of maltreatment: study protocol for a randomised controlled trial
- Corrected title:
  Trial of healthy relationship initiatives for the very early years (THRIVE), evaluating Enhanced Triple P for Baby and Mellow Bumps **for those with** additional social and care needs during pregnancy and their infants who are at higher risk of maltreatment: study protocol for a randomised controlled trial

The original article has been corrected.
