# Supplementary material for: Trial of healthy relationship initiatives for the very early years (THRIVE), evaluating Enhanced Triple P for Baby and Mellow Bumps additional social and care needs during pregnancy and their infants who are at higher risk of maltreatment: study protocol for a randomised controlled trial
Source: Trials. 2019 Aug 14;20:499. doi: 10.1186/s13063-019-3571-5 (PMC6694522; doi:10.1186/s13063-019-3571-5)
Supplement: Supplementary file 1 — Information sheet and consent form (v3.0 06.09.17). Information sheet provided to participants before study participation and consent form to be completed by all enrolled participants. (PDF 454 kb) [file 13063_2019_3571_MOESM1_ESM.pdf]

INFORMATION SHEET and CONSENT FORM (Recruit to trial)

The THRIVE Trial is funded by the National Institute for Health Research's Public Health Research Programme.

Participant information sheet (recruitment)

We would like to invite you to take part in the THRIVE study. Before you decide whether or not to take part we would like you to understand why you have been asked to take part and what taking part involves.

What is the study about?

There are a number of parenting support groups running in Scotland that are designed to improve the health and wellbeing of mothers and their babies. The THRIVE study aims to find out if two such groups, Enhanced Triple P for Baby (ETPB) and Mellow Bumps (MB) benefit mothers by improving their wellbeing and the relationship between them and their baby compared to current treatments being offered. We are also interested in finding out if one group is more helpful to mothers and their child and if the benefits carry on as the infant grows. This kind of research may help health practitioners to offer the right kind of help to give mothers and their babies the best start in life.

Why have I been asked to take part?

You have been asked to take part in this study because you are currently pregnant and your midwife suggested that you might be interested in helping us with our research.

What would I have to do if I took part in the study?

If you were interested in taking part in the study one of our researchers would meet with you, at your home (or a different location of your choice), so that they could answer any questions you have and to ask you to complete a consent form. You will then be asked to complete the first questionnaire of the study. This visit should take about 1 hour and in order to thank you for your time we will offer you a £15 voucher so that you can buy something for yourself or your baby.

You will be contacted again at the following times:

1. Within 8 weeks of completing the first questionnaire to tell you which group you will be taking part in. There are three groups; Enhanced Triple P for Baby, Mellow Bumps or routine maternity care. You cannot choose which group you will be in. If you are in the Enhanced Triple P for Baby or Mellow Bumps groups you will continue to receive your routine maternity care. We will cover of the cost of the group and arrange for a taxi to take you to and from the venue. If you are in the routine maternity care group you will receive your routine maternity care alone and will not take part in Enhanced Triple P for Baby or Mellow Bumps sessions.
2. For the Enhanced Triple P for Baby or Mellow Bump groups you will contacted after your baby is 6 weeks old in order to arrange the postnatal part(s) of the groups. You may also be asked to take part in a telephone interview to discuss your experiences of the postnatal part of the groups. These telephone interviews should take no more than 30 mins.
3. When your baby is around 6 months old we would like to take a short video recording (15 minutes) of you and your baby together, perhaps when you are playing together. You will be asked to fill out a questionnaire similar to the one you did before, it will also include questions on how you have found being a parent. This visit will take about 1 hour and in order to thank

you for your time we will offer you a £15 voucher so that you can buy something for yourself or your baby.

4. You may also be asked to take part in one-to-one interviews to discuss your experience of taking part in the study with a member the research team. The interviews will take place at different points throughout your involvement in the trial. Interviews will be conducted face-to-face in your home or another location of your choice. The interview(s) will take no more than 1 hour and in order to thank you for your time we will offer you a £15 voucher so that you can buy something for yourself or your baby

What are the benefits of the study?

By taking part in this study you will be helping to identify which of the two support groups benefit mothers and their babies the most so that in the future women can get the right kind of help to be the best mothers they can be.

Is there a downside to taking part?

We do not expect that taking part will cause you any distress. If it happens we could contact your midwife or Doctor to help you find the support that you need if you ask us to.

Do I have to take part?

No. Taking part in the study is voluntary. The care and support that you receive from your midwife and any other services will not change in any way if you choose not to take part.

What if I'm not asked to attend one of the groups?

Some women will be asked to participate in the study without attending one of the groups. This is because <u>we want to learn whether or not these groups can help mothers and babies</u>. If you are asked to take part without being in a group you play a very important part in the study as we will be able to test if these groups benefit women compared to routine maternity care.

Why do you want to access records for myself and my baby?

We would like to access your own and your child's health, education, social care and justice records, such as maternity records and your child's school attendance, to spot if there are any long-term benefits from taking part in the study. This would involve looking at information that is collected by professionals as part of you and your baby's normal care such as the weight of your baby along with information about the services you use after your baby is born. We would also like to find out how your baby grows by looking at the health information that is collected routinely as children grow up including information from Social Work, Education and Justice Departments.

Your baby will have a routine blood test (called the Guthrie or 'heel prick' test) when they are about 5 days old. This is taken from their heel onto a card. These cards are routinely kept by the NHS for a number of years. The dried blood spots contain a number of chemicals and proteins that can be useful in finding out about causes of disease. We would like your permission to use blood that is left-over from this test for research in the future. You would not need to meet with a researcher again and we would not contact you about the information that we access. The researchers who would see the information have a duty of care to you and will not discuss you with anyone outside of the study.

Finally, we would like to access routine information in order to obtain up to date contact details for yourself in case you change your number or move house during the length of time you are involved in the trial.

What kind of information about me and my child would you like to look at?

Taking part in one of the groups may help mothers and their babies to lead a healthy and full life. We would like to use your and your baby's date of birth and Community Health Index (CHI) number to access and link information that is held by different organisations. This information along with things that you tell us when we meet you will help us to find out whether the groups have helped the women who took part. A CHI number is allocated to everyone registered with a GP in Scotland.

Information from routine health records

The National Health Service (NHS) keeps information on everyone accessing health services through routine medical and other health-related records. These records are held within health databases, which may record information about:

- Admissions or attendances at hospital (including diagnoses received and treatments given)
- Visits to your family doctor or other health professionals, including any medicines prescribed
- Records of immunisations and health checks

We would like to access information from these routine and other health related records about you and your baby. This would mean that we would have a much more complete picture of your health.

Information from routine education records

We would like to collect information from routine records on education about your child's performance at school. By performance at school, we mean the results that they get when they do national tests or the formal assessments that teachers make. The exact information varies depending on where you live but we mean things like 5-14 levels and Standard grades in Scotland. We would like to get the information from these routine education records about your child from the start of pre-school education up to the end of their education. This information will help us to have a more complete picture of your child's development.

Information from social care records

We would like to collect information from social care records about your situation and the help that you may have received. We would like to obtain the information from these records about you and your baby. This may include letters, notes and reports about the support provided for you and your baby and about the help that you both need.

This information will help us to have a more complete picture of your situation and the circumstances that your child is growing up in.

Information from criminal justice services

We would like to collect information from criminal justice services about your situation. We would like to obtain the information from these records about you and your child. This may include letters and reports used in court proceedings. This information will help us to have a more complete picture of your situation and your child's circumstances as they grow up.

Why do you need to notify my G.P. or social worker about my participation in the study?

We would like to let your G.P. or social worker know that you are taking part in the study so they can understand what sort of support you are getting in your pregnancy. We may also contact your G.P. or social worker if we lose touch with you during the study in order to get new contact details for you. Most importantly we would like to be able to contact your G.P. or social worker if we are worried about you or your baby and think you would benefit from their help.

The THRIVE Trial is funded by the National Institute for Health Research's Public Health Research Programme.

What if I agree to take part but then change my mind?
You can stop taking part in the study at any time, and more importantly, you don't have to tell us why. We will destroy information that you could be identified from but we will continue to use the data collected up until you stopped taking part. Your decision to stop taking part in the study will not affect the care that you or your baby receives.

How will the results be used?
We hope to find that Enhanced Triple P for Baby and Mellow Bumps improve the wellbeing of both mother and baby. We also hope to find that one of the groups will be better at doing this than the other and that it will be of more benefit to mothers' than the usual treatment provided by the NHS. This could lead to improvements in services provided to mothers in the future.

What will happen to the information I supply?
The answers you give will be completely anonymous. However, if we feel that you or your baby is at risk then we will need to tell the health care professionals caring for you. All data will be stored in a locked filing cabinet accessed only by members of the research team and approved persons to check that the study is being carried out correctly. All of these people have a duty of confidentiality to you as a participant and they will not discuss your information with anyone who is not involved in the study. Your personal contact details will be stored separately from your other answers, also in locked storage.

The video footage taken of you and your baby will also be stored in locked filing cabinets and will only be viewed by members of the research team. An external examiner will only look at the video footage to make sure that we are analysing it correctly. At the end of the study all of the video-tapes will be destroyed.

If you become upset during a group session, video-taping or a telephone consultation, the researcher will discuss the possible involvement of your care professional, GP or Health Visitor with you. With your consent, she may contact these health care professionals on your behalf.

What will happen when the study is complete?
You will be involved in the study for approximately18 months and the study is scheduled to be completed by July 2019. All of the information that you give the researchers will be kept safely by the University of Glasgow or by one of our research partners, at the University of Manchester, Glasgow Caledonian, University, the University of Newcastle or the University of Aberdeen. If you give your consent it may be used by other researchers with the University of Glasgow's approval, under the strict rules governing the confidentiality of your information. Your name or any material that might identify you or your baby will never be used or given to anyone.

Once all the data has been collected and analysed, the findings will be written in reports to be published in academic journals and the findings will be presented at conferences. A summary report of the findings will be available for participants at the end of the study.

What if there is a problem?
If you have a concern about any aspect of this study, you should ask to speak to the researchers who will do their best to answer your questions. If they are unable to help you or you wish to make a complaint about the study, please contact the Institute of Health and Wellbeing, University of Glasgow, Research Support Manager by phone on 0141 353 7500 or by email at survadmin@sphsu.mrc.ac.uk.

The THRIVE Trial is funded by the National Institute for Health Research's Public Health Research Programme.

In the unlikely event that something does go wrong and you are harmed during the research you may have grounds for a legal action for compensation against the University of Glasgow, but you may have to pay your legal costs. The normal National Health Service (NHS) complaints mechanisms will still be available to you. You can contact 0141 201 4500 for Greater Glasgow and Clyde NHS Complaints Team and 01292 513 620 for Ayrshire and Arran NHS Complaints Team.

Who is involved in the research?

The research is led by Dr. Marion Henderson, and involves researchers based at the University of Glasgow, Glasgow Caledonian University, the University of Aberdeen, the University of Manchester and the University of Newcastle. During the study you will be contacted by researchers based at either the University of Glasgow or Glasgow Caledonian University. You can contact the THRIVE team by telephone on 0800 389 2129 or by emailing sphsu-thrive@glasgow.ac.uk

Who has reviewed the study?

This study has been reviewed by the West of Scotland NHS Research Ethics Committee.

What do I do now?

If you have decided that you would like to take part, you will be asked to sign a consent form. You will then be given the initial questionnaires to complete.

Thank you for considering taking part in this study

Dr. Marion Henderson
Chief Investigator for THRIVE

The THRIVE Trial is funded by the National Institute for Health Research's Public Health Research Programme.

CONSENT FORM: Participation in the THRIVE trial

(PARTICIPANT COPY)

Participant ID: ………………………… Name of Researcher: ……..........……………………....

PLEASE READ EACH OF THE STATEMENTS BELOW, INITIAL EACH BOX AND SIGN AT THE BOTTOM IF YOU AGREE

Please initial each box

I confirm that I have read and understand the information sheet version 3.0, dated 06.09.17	☐
I have had the chance to ask questions about the study and I am happy with the answers that I have been given	☐
I understand that the study will involve:	
• completing a questionnaire at the start of the study and when my baby is 6 months old	☐
• being video-recorded caring for my baby when he/she is 6 months old	☐
• perhaps being asked to participate in one-to-one interviews with a member of the research team about my experience of taking part in the study	☐
If I am in a group receiving Enhanced Triple P for Baby or Mellow Bumps I agree to:	
• a member of the research team being present during the group session	☐
• being audio or video-recorded during the sessions, but only if <u>all</u> group members agree to this	☐
• complete two short questionnaires on my expectations and experience of taking part in the group sessions	☐
• complete a short questionnaire after each session	☐
• perhaps being asked to take part in a 20-30 minute phone interview about my experiences of going to the groups	☐

The THRIVE Trial is funded by the National Institute for Health Research's Public Health Research Programme.

I give permission for my partner to be asked to complete a questionnaire and be invited to participate in a one-to-one interview with a member of the research team	☐
I understand that I do not need to take part in the study and that I can say “no” or change my mind at any time without giving any reason and without my normal antenatal care being affected	☐
If I decide that I can no longer take part in the study I agree to be contacted by the research team about my reasons for no longer wanting to take part	☐
I understand that taking part in the study will not affect the care that I or my baby receive from health and social care professionals	☐
I agree that the research team can tell my GP, Midwife, Health Visitor and Social Worker of my participation in this study	☐
I agree that the research team can contact my Health Professional to ask about the outcome of my pregnancy as well as my use of services	☐
I agree to both mine and my baby’s data being retained by the research team and used for the study if I decide that I no longer want to take part in the study	☐
I understand that the information collected in the study will be used in research reports and articles but that my name will not be used	☐
I understand that things I say in the interview may be quoted in research reports and articles without using my name	☐
I understand that any information I provide will be private and will not be seen by anyone outside of the University of Glasgow and their researcher partners in the THRIVE trial. The information will be stored securely and destroyed according to University of Glasgow best research practice guidelines	☐
I agree to take part in the study	☐

I have read, understand and completed this form

______________________	__________	______________________
**Participant signature**	**Date**	**Name in block capitals**

The THRIVE Trial is funded by the National Institute for Health Research's Public Health Research Programme.

Researcher signature	Date	Name in block capitals

The THRIVE Trial is funded by the National Institute for Health Research's Public Health Research Programme.

THE NEXT QUESTIONS RELATE TO ADDITIONAL RESEARCH THAT MAY HAPPEN IN THE FUTURE

Participant ID: ……………...Name of Researcher:…………………………….................

PLEASE READ EACH OF THE STATEMENTS BELOW, INITIAL EACH BOX AND SIGN AT THE BOTTOM IF YOU AGREE

Permission to release information from routine records

I agree that information from my routine records can be released to the THRIVE study ☐

Please place a tick in the boxes to say whether or not you give permission for:

Health	Yes	☐	No	☐
Education	Yes	☐	No	☐
Social care	Yes	☐	No	☐
Criminal justice services	Yes	☐	No	☐

Parental permission to release information from routine records

I agree that information from routine records held, from birth to age 16 years, for my child(ren) can be released to the THRIVE study ☐

Please place a tick in the boxes to say whether or not you give permission for:

Health	Yes	☐	No	☐
Education	Yes	☐	No	☐
Social care	Yes	☐	No	☐
Criminal justice services	Yes	☐	No	☐

The THRIVE Trial is funded by the National Institute for Health Research's Public Health Research Programme.

I understand that the information obtained from mine and my baby's records will be treated in strict confidence in accordance with the Data Protection Act and used for research purposes only.	☐
I agree that researchers may use left-over blood taken for the baby's new born spot screening test, which is routinely kept by the NHS, in future research.	☐
I understand and agree that information related to me and my baby may be made available to other researchers in the future for further research, but that this would be overseen by the University of Glasgow and no information that could identify me or my baby (e.g. names and addresses) would be given to these researchers	☐
I agree to be contacted by the research team about the study after the THRIVE trial has finished	☐
I agree that researchers from the from the University of Glasgow may access my routine Health, Education, Social or Criminal Justice data in order to obtain up-to-date contact details for me throughout my time in the trial.	☐

I have read, understand and completed this form

______________________	__________	______________________
**Participant signature**	**Date**	**Name in block capitals**

______________________	__________	______________________
**Researcher signature**	**Date**	**Name in block capitals**

The THRIVE Trial is funded by the National Institute for Health Research's Public Health Research Programme.

PERMISSION TO USE COMMUNITY HEALTH INDEX (CHI) NUMBER

Participant ID: ……………… Name of Researcher:……………………………........

I agree that the research team can access my routine health records through my Community Health Index (CHI) number which will be supplied by my health professional	☐
I agree that the research team can access my baby's routine health records through his/her Community Health Index (CHI) number which will be supplied by my health professional	☐

I have read, understand and completed this form

____________________	__________	____________________
**Participant signature**	**Date**	**Name in block capitals**
____________________	__________	____________________
**Researcher signature**	**Date**	**Name in block capitals**

The THRIVE Trial is funded by the National Institute for Health Research's Public Health Research Programme.

CONSENT FORM: Participation in the THRIVE trial

(RESEARCHERS COPY: PLEASE TEAR OUT)

Participant ID: …………………….Name of Researcher:……………………………......

PLEASE READ EACH OF THE STATEMENTS BELOW, INITIAL EACH BOX AND SIGN AT THE BOTTOM IF YOU AGREE

Please initial each box

I confirm that I have read and understand the information sheet version 3.0, dated 06.09.17	☐
I have had the chance to ask questions about the study and I am happy with the answers that I have been given	☐
I understand that the study will involve:	
• completing a questionnaire at the start of the study and when my baby is 6 months old	☐
• being video-recorded caring for my baby when he/she is 6 months old	☐
• perhaps being asked to participate in one-to-one interviews with a member of the research team about my experience of taking part in the study	☐
If I am in a group receiving Enhanced Triple P for Baby or Mellow Bumps I agree to:	
• a member of the research team being present during the group session	☐
• being audio or video-recorded during the sessions, but only if all group members agree to this	☐
• complete two short questionnaires on my expectations and experience of taking part in the group sessions	☐
• complete a short questionnaire after each session	☐
• perhaps being asked to take part in a 20-30 minute phone interview about my experiences of going to the groups	☐
I give permission for my partner to be asked to complete a questionnaire and be invited to participate in a one-to-one interview with a member of the research team	☐

The THRIVE Trial is funded by the National Institute for Health Research's Public Health Research Programme.

I understand that I do not need to take part in the study and that I can say “no” or change my mind at any time without giving any reason and without my normal antenatal care being affected	☐
If I decide that I can no longer take part in the study I agree to be contacted by the research team about my reasons for no longer wanting to take part	☐
I understand that taking part in the study will not affect the care that I or my baby receive from health and social care professionals	☐
I agree that the research team can tell my GP, Midwife, Health Visitor and Social Worker of my participation in this study	☐
I agree that the research team can contact my Health Professional to ask about the outcome of my pregnancy as well as my use of services	☐
I agree to both mine and my baby’s data being retained by the research team and used for the study if I decide that I no longer want to take part in the study	☐
I understand that the information collected in the study will be used in research reports and articles but that my name will not be used	☐
I understand that things I say in the interview may be quoted in research reports and articles without using my name	☐
I understand that any information I provide will be private and will not be seen by anyone outside of the University of Glasgow and their researcher partners in the THRIVE trial. The information will be stored securely and destroyed according to University of Glasgow best research practice guidelines	☐
I agree to take part in the study	☐

I have read, understand and completed this form

______________________	__________	______________________
**Participant signature**	**Date**	**Name in block capitals**
______________________	__________	______________________
**Researcher signature**	**Date**	**Name in block capitals**

The THRIVE Trial is funded by the National Institute for Health Research's Public Health Research Programme.

THE NEXT QUESTIONS RELATE TO ADDITIONAL RESEARCH THAT MAY HAPPEN IN THE FUTURE

Participant ID: …………….......... Name of Researcher:……………………………........

PLEASE READ EACH OF THE STATEMENTS BELOW, INITIAL EACH BOX AND SIGN AT THE BOTTOM IF YOU AGREE

Permission to release information from routine records

I agree that information from my routine records can be released to the THRIVE study ☐

Please place a tick in the boxes to say whether or not you give permission for:

Health	Yes	☐	No	☐
Education	Yes	☐	No	☐
Social care	Yes	☐	No	☐
Criminal justice services	Yes	☐	No	☐

Parental permission to release information from routine records

I agree that information from routine records held, from birth to age 16 years, for my child(ren) can be released to the THRIVE study ☐

Please place a tick in the boxes to say whether or not you give permission for:

Health	Yes	☐	No	☐
Education	Yes	☐	No	☐
Social care	Yes	☐	No	☐
Criminal justice services	Yes	☐	No	☐

The THRIVE Trial is funded by the National Institute for Health Research's Public Health Research Programme.

I understand that the information obtained from mine and my baby's records will be treated in strict confidence in accordance with the Data Protection Act and used for research purposes only.	☐
I agree that researchers may use left-over blood taken for the baby's new born spot screening test, which is routinely kept by the NHS, in future research.	☐
I understand and agree that information related to me and my baby may be made available to other researchers in the future for further research, but that this would be overseen by the University of Glasgow and no information that could identify me or my baby (e.g. names and addresses) would be given to these researchers	☐
I agree to be contacted by the research team about the study after the THRIVE trial has finished	☐
I agree that researcher from the from the University of Glasgow may access my routine Health, Education, Social or Criminal Justice data in order to obtain up-to-date contact details for me throughout my time in the trial.	☐

I have read, understand and completed this form

____________________	__________	____________________
**Participant signature**	**Date**	**Name in block capitals**
____________________	__________	____________________
**Researcher signature**	**Date**	**Name in block capitals**

The THRIVE Trial is funded by the National Institute for Health Research's Public Health Research Programme.

PERMISSION TO USE COMMUNITY HEALTH INDEX (CHI) NUMBER

Participant ID: ……………………….. Name of Researcher: ……………………………..

I agree that the research team can access my routine health records through my Community Health Index (CHI) number which will be supplied by my health professional	☐
I agree that the research team can access my baby's routine health records through his/her Community Health Index (CHI) number which will be supplied by my health professional	☐

I have read, understand and completed this form

______________________	__________	______________________
**Participant signature**	**Date**	**Name in block capitals**
______________________	__________	______________________
**Researcher signature**	**Date**	**Name in block capitals**

The THRIVE Trial is funded by the National Institute for Health Research's Public Health Research Programme.

The THRIVE Trial is funded by the National Institute for Health Research's Public Health Research Programme.
