# Supplementary material for: Trial of healthy relationship initiatives for the very early years (THRIVE), evaluating Enhanced Triple P for Baby and Mellow Bumps additional social and care needs during pregnancy and their infants who are at higher risk of maltreatment: study protocol for a randomised controlled trial
Source: Trials. 2019 Aug 14;20:499. doi: 10.1186/s13063-019-3571-5 (PMC6694522; doi:10.1186/s13063-019-3571-5)
Supplement: Supplementary file 2 — THRIVE baseline questionnaire (v1.5 15.11.13). Questionnaire completed by study participants at the baseline visit (12–24 weeks gestation) (PDF 5336 kb) [file 13063_2019_3571_MOESM2_ESM.pdf]

Trial of **H**ealthy **R**elationship **I**nitiatives for the **V**ery **E**arly-years

Baseline Questionnaire

For administration purposes only

Date: ☐☐ ☐☐ ☐☐☐☐

Participant ID: ______________________

Researcher ID: ______________________

Circle as appropriate: **SC / SCHR / RC**

Randomisation **Y / N**

Supplementary sheet **Y / N**

PLACE BARCODE HERE
CHECK MATERIAL CODE CORRECT

Your answers will remain confidential and will not be seen by:

- **your family or friends**
- **your health or social care practitioner**

so please be honest about how you feel and what you think.

There are no 'right' or 'wrong' answers. We just want to know what you think and something about your experiences.

Please read the instructions carefully.

If you are not sure what a question means please ask the researcher.

If you do not want to answer a question, please just leave it blank and go on to the next question.

Please detach this page and give it to the researcher who will file it separately from the rest of the questionnaire.

What is your date of birth?
(Please **write in** - for example: 30/03/1983)

D D / M M / Y Y Y Y

Do you know your postcode? If so, tick 'yes' and write it down, if no tick 'no'. If you only know the beginning then please write this in.

☐ 1 **Yes**, my postcode is: ☐☐☐☐ ☐☐☐

e.g. K A 1 4 8 R J

G 1 2 8 R Z

☐ 2 **No**

PLACE BARCODE HERE
CHECK MATERIAL CODE CORRECT

Blank page

About you

1	**Which religion are you?** (Please tick **all that apply**)

None ☐ 1
Protestant ☐ 1
Roman Catholic ☐ 1
Other Christian (Please **write in**) ☐ 1
____

Buddhist ☐ 1
Hindu ☐ 1
Jewish ☐ 1
Muslim ☐ 1
Sikh ☐ 1
Other (Please **write in**) ☐ 1
____

2	**Do you go to church or to another place of worship?** (Please tick **one box only**)

Yes, at least once a week ☐ 1
Yes, at least once a month ☐ 2
Yes, at least once a year ☐ 3
No, not at all ☐ 4

3	**Which ethnic background do you belong to?** (Please tick **all that apply**)

White

- Scottish ☐ 1
- British ☐ 1
- English ☐ 1
- Irish ☐ 1
- Northern Irish ☐ 1
- Welsh ☐ 1
- Any other White background (Please **write in**) ☐ 1

____

Asian or Asian British

- Bangladeshi ☐ 1
- Indian ☐ 1
- Pakistani ☐ 1
- Any other Asian background (Please **write in**) ☐ 1

____

Black or Black British

- African ☐ 1
- Caribbean ☐ 1
- Any other Black background (Please **write in**) ☐ 1

____

4	**How old were you when you left secondary school?** (Please **write in**)

______________________ Years old

5	**What is your highest educational qualification?**<br>(Please tick **one box only**)

No educational qualifications	☐	1
Standard Grades, Intermediate 1 or 2, O Grades, O Levels, GCE/GCSEs	☐	2
Higher, Advanced Higher, A levels	☐	3
Vocational qualification (e.g. Access, SVQ, SCOTVEC, BTEC)	☐	4
HNC/HND	☐	5
Undergraduate Degree (e.g. BA/BSc)	☐	6
Postgraduate qualification (e.g. MSc, PhD)	☐	7
Other<br>(Please **write in**)	☐	8

____

6	**Which of these statements best describes you?**<br>(Please tick **one box only**)

I am currently in paid employment or self-employed	☐	1	**Go to Question 7**
I am not currently working but have been in paid employment in the past	☐	2	**Go to Question 8**
I have never been in paid employment	☐	3	**Go to Question 11**

7	**How many hours did you work last week?**<br>(Please **write in**)

____________________ Hours

8	**Please tell us about your current job or your last job if you are not working.** (Please **write in**)
**Job title** Example: waitress	
**What this actually involves** Example: taking food orders, serving customers food and drink	
**Employer type** Example: restaurant	

9	**Which of these best describes your current job or your last job if you are not working now?** (Please tick **one box only**)

Self employed with paid employees	☐	1
Self employed with NO paid employees	☐	2
Manager	☐	3
Supervisor	☐	4
Employee	☐	5
Don't know	☐	6

10	**What size of company is/was it?** (Please tick **one box only**)

Under 25 staff	☐	1
25 staff or more	☐	2
Don't know	☐	3

11	**Which describes best what you were doing last week?** (Please tick **one box only**)

In paid employment ☐ 1

Doing unpaid work for a business that you own or that a relative owns ☐ 2

Waiting to take up paid work already obtained ☐ 3

On a Government scheme for employment training ☐ 4

Looking for paid work on a Government training scheme ☐ 5

Intending to look for work but prevented by temporary sickness or illness ☐ 6

Permanently unable to work because of long-term sickness or disability ☐ 7

At college or university full time ☐ 8

At college or university part time ☐ 9

In full time secondary education (e.g. attending high school) ☐ 10

Retired from paid work ☐ 11

Looking after home or family ☐ 12

Providing full time care for an ill or disabled friend or relative ☐ 13

Doing something else
(Please **write in**) ☐ 14

____

____

12	**I am currently living...** (Please tick **all that apply**)

in a house or flat that is owned outright ☐ 1

in a house or flat that is being bought with the help of a mortgage or loan ☐ 1

in a house or flat rented from a council, local authority or housing association ☐ 1

in a house or flat rented from a private landlord ☐ 1

at home with my parents ☐ 1

rent free with a family member or friend ☐ 1

in a hostel, bed and breakfast, homeless shelter or temporary accommodation ☐ 1

in a children's unit, foster care placement or supported care placement ☐ 1

other (please **describe**) ☐ 1

____

____

13	**How many rooms are there in your home, excluding the kitchen and bathroom?** (Please **write in**)

____

14	**How much of a problem do you have with damp, mould or condensation on the walls in your home, apart from in the kitchen or bathroom?** (Please tick **one box only**)

None, there is no damp ☐ 1 Not much of a problem ☐ 2

Some problem ☐ 3 Great problem ☐ 4

15	**In your home, which of these things do you have in working order?** (Please tick **all that apply**)

Fridge	☐ 1	Dishwasher	☐ 1
Freezer	☐ 1	DVD/Blu ray player	☐ 1
Washing machine	☐ 1	Tumble dryer	☐ 1
Microwave	☐ 1	Landline telephone	☐ 1
Television	☐ 1	Cable or Satellite TV (e.g. Sky, Virgin, BT TV)	☐ 1
Broadband internet access	☐ 1	I don't own any of these items	☐ 1

16	**How many cars and/or vans do the people living in your house own?** (Please tick **one box only**)

None	☐ 1	One	☐ 2
Two	☐ 3	Three or more	☐ 4

17	**During the past 12 months how many times did you travel away from home (including UK trips) on holiday?** (Please tick **one box only**)

Not at all	☐ 1	Once	☐ 2
Twice	☐ 3	Three or more	☐ 4

18 How many people in total (including yourself and all children of all ages) live here most of the time as members of this household?

__________ Persons

For each member of the household, excluding you, could you tell me:

	Their relationship to you (e.g. partner, daughter, son or friend)	Their relationship to your baby (e.g. father, sister or grandparent)	Sex M	Sex F	Age	Pre-school	School	College/ university	At work /training	Unemployed	Retired	House-person	Other
						**What do they do?** (Please tick **one box only**)							
**Example 1**	*Partner*	*Father*	✓ 1	2	*28*	1	2	3	4	✓ 5	6	7	8
**Example 2**	*Daughter*	*Sister*	1	✓ 2	*6*	1	✓ 2	3	4	5	6	7	8
			1	2		1	2	3	4	5	6	7	8
			1	2		1	2	3	4	5	6	7	8
			1	2		1	2	3	4	5	6	7	8
			1	2		1	2	3	4	5	6	7	8
			1	2		1	2	3	4	5	6	7	8
			1	2		1	2	3	4	5	6	7	8
			1	2		1	2	3	4	5	6	7	8
			1	2		1	2	3	4	5	6	7	8
			1	2		1	2	3	4	5	6	7	8

Your childbirth history

19	**Is this your first pregnancy?** (Please tick **one box only**)

Yes ☐ 1 — **Go to Question 25**

No ☐ 2 — **Go to Question 20**

20	**Have you ever had...** (Please tick **one box per line**)

	**Yes**	**No**
a miscarriage?	☐ 1	☐ 2
an abortion or termination?	☐ 1	☐ 2

21	**How old were you when you first became pregnant?** (Please **write in**)

________________ Years

22	**How many times have you been pregnant, including this pregnancy?** (Please **write in**)

________________ Times

23	Did any of the following happen during any of your previous pregnancies? (Please tick **one box per line**)	
	**Yes**	**No**
I experienced anxiety or depression	☐ 1	☐ 2
I regularly drank alcohol to the point of drunkenness	☐ 1	☐ 2
I regularly used illegal (street) drugs	☐ 1	☐ 2
I was prescribed an opiate substitute (e.g. methadone or buprenorphine (Subutex/Suboxone))	☐ 1	☐ 2
My partner/the father of my baby was abusive to me	☐ 1	☐ 2
I had a social worker	☐ 1	☐ 2
I was homeless for all or part of my pregnancy	☐ 1	☐ 2
I experienced complications that required medication	☐ 1	☐ 2
I experienced complications that required a stay in hospital	☐ 1	☐ 2
I experienced complications during the birth/labour	☐ 1	☐ 2
My child was born prematurely	☐ 1	☐ 2
My child was kept in hospital after birth for medical treatment	☐ 1	☐ 2
My child died during pregnancy, was stillborn or died shortly after birth	☐ 1	☐ 2
My child was removed by social work services at birth	☐ 1	☐ 2
My child was removed by social work services before their first birthday	☐ 1	☐ 2

24 **For each pregnancy that resulted in a live birth, we would like to ask you some questions.**
(Please fill out **one line for each child** you have given birth to)

		Sex		Was this child born before 37 weeks gestation?		Did this child weigh less than 2.5 Kg (5 lbs 8 oz) when born?	
**Child**	**Year of birth**	M	F	Yes	No	Yes	No
**1**		☐ 1	☐ 2	☐ 1	☐ 2	☐ 1	☐ 2
**2**		☐ 1	☐ 2	☐ 1	☐ 2	☐ 1	☐ 2
**3**		☐ 1	☐ 2	☐ 1	☐ 2	☐ 1	☐ 2
**4**		☐ 1	☐ 2	☐ 1	☐ 2	☐ 1	☐ 2
**5**		☐ 1	☐ 2	☐ 1	☐ 2	☐ 1	☐ 2

This pregnancy

25	**Have you been told your expected "due date"? This is the estimated date that you will give birth to your baby.** (Please tick **one box only**)
	Yes ☐ 1 **Go to Question 26**
	No ☐ 2 **Go to Question 27**

26	**What is your expected due date?** (Please **write in** - for example: 15/03/2014)
	D D / M M / Y Y Y Y

27	**How many babies have you been told that you are having?** (Please tick **one box only**)
	One ☐ 1 Two ☐ 2 Three ☐ 3 Four or more ☐ 4

28	**Other than your midwife or other medical professionals, who do <u>you</u> want to be with you when you are having the baby?** (Please tick **all that apply**)
	The baby's father ☐ 1
	My current partner (if different from baby's father) ☐ 1
	My Mum ☐ 1
	My Dad ☐ 1
	Another family member ☐ 1
	My friend ☐ 1
	I do not feel that I have anyone to support me ☐ 1
	I do not want anyone to support me ☐ 1
	Other (please **write in**, e.g. key worker/social worker) ☐ 1
	______________________________

29	**Where would you like to give birth to your baby?** (Please tick **one box only**)

At home ☐ 1

In a midwifery led unit ☐ 2

In a consultant led unit at a hospital ☐ 3

I haven't decided yet ☐ 4

30	**Some pregnancies are planned and others are a surprise. Which of these best describes your pregnancy?** (Please tick **all that apply**)

We both hoped it would happen ☐ 1

I hoped it would happen ☐ 2

My partner/the father of my baby hoped it would happen ☐ 3

It wasn't planned ☐ 4

31	**Which of the following have you felt since you became pregnant?** (Please circle **all that apply**)

Protected	Unsure	Happy
Not bothered	Excited	Anxious
Resentful	Irritable	Relaxed
Loving	Worried	Supported
Angry	Confused	Calm
Don't care	Unhappy	Protective
Laid-back	Serious	Proud
Scared	Caring	Nervous
Strong	Other	Weak

↓

____

(Please **write in**)

32	**The following questions are about how you have been feeling during the past month.** (Please tick **one box per line**)

	Not at all	A little	A lot	Very much
Have you been worrying that you might not be a good mother?	1	2	3	4
Have you been worrying about hurting your baby inside you?	1	2	3	4
Has it worried you that you may not have any time to yourself once your baby is born?	1	2	3	4
Have you been feeling happy that you are pregnant?	1	2	3	4
Has the thought of having more children appealed to you?	1	2	3	4
Have you been looking forward to caring for your baby's needs?	1	2	3	4
Have you been wondering whether your baby will be healthy and normal?	1	2	3	4
Have you felt that life will be more difficult after the baby is born?	1	2	3	4
Has the thought of breastfeeding your baby appealed to you?	1	2	3	4

	Very much	A lot	A little	Not at all
Has the thought of wearing maternity clothes appealed to you?	4	3	2	1
Have you felt that pregnancy was unpleasant?	4	3	2	1

	Never	Rarely	Often	Very often
Have you regretted being pregnant?	1	2	3	4

Your health and wellbeing

33 **Thinking about your pregnancy so far, would you say that you have generally been ...**
(Please tick **one box only**)

very well	fairly well	not very well	not well at all
☐ 1	☐ 2	☐ 3	☐ 4

34 **Which of these statements best describes you?**
(Please tick **one box only**)

Smoker	☐ 1	**Go to Question 36**
Ex-smoker	☐ 2	**Go to Question 35**
Non-smoker	☐ 3	**Go to Question 37**

35 **How long has it been since you stopped smoking?**
(Please **write in**, if you're not sure then please write in your best guess)

__________ **years** __________ **months** __________ **days**

36 **On average how many cigarettes did you smoke per day ...**
(Please tick **one box per column**)

	**before you found out you were pregnant?**	**since you found out you were pregnant?**
None	☐ 1	☐ 1
10 or less	☐ 2	☐ 2
11 – 20	☐ 3	☐ 3
21 – 30	☐ 4	☐ 4
More than 30	☐ 5	☐ 5

37	**How often did you drink until you felt drunk...** (Please tick **one box per column**)	
	**before you found out you were pregnant?**	**since you found out you were pregnant?**
Every day per week	☐ 1	☐ 1
A few days per week	☐ 2	☐ 2
A few days per month	☐ 3	☐ 3
Once or more per month	☐ 4	☐ 4
Never	☐ 5	☐ 5

38	**Why do you drink alcohol?** (Please circle **all that apply**)	
To relax	To forget	To have a good time
To numb pain	To de-stress	Boredom
I like the taste	Everyone does it	To feel more confident
My family/friends tell me to	Habit	To get drunk

I do not drink alcohol (please **tick**) ☐ 1

39	**Have you ever smoked cannabis?** (Please tick **all that apply**)
No	☐ 1
Yes, in the past	☐ 1
Yes, during this pregnancy	☐ 1

40	**Have you ever taken any other illegal (street) drug? (e.g. heroin, crack/cocaine, valium, ecstasy)** (Please tick **all that apply**)

No	☐	1	**Go to Question 44**
Yes, in the past	☐	1	**Go to Question 41**
Yes, during this pregnancy	☐	1	**Go to Question 41**

41	**Have you ever injected any illegal (street) drug? (e.g. heroin, crack/cocaine, temazepam, amphetamines)** (Please tick **all that apply**)

No	☐	1
Yes, in the past	☐	1
Yes, during this pregnancy	☐	1

42	**Are you currently being prescribed an opiate substitute drug? (e.g. methadone or buprenorphine (Subutex/Suboxone))** (Please tick **one box only**)

No	☐	1	**Go to Question 44**
Methadone	☐	2	**Go to Question 43**
Subutex/Suboxone	☐	3	**Go to Question 43**

43	**Are you using opiate substitute drugs that are not prescribed to you? (e.g. street methadone or buprenorphine (Subutex/Suboxone))** (Please tick **one box only**)

No	☐	1
Yes, I am buying it from someone	☐	2
Yes, I have been prescribed an opiate substitute **and** I am also buying it from someone	☐	3

44 **The following questions are about your substance use. Please answer these questions about one substance you have used in the last month (for example: cigarettes/alcohol/cannabis/heroin/crack).**

If you don't smoke cigarettes, drink alcohol or take any illegal substances please tick the box below

☐ 1 **then go to Question 45**

Which substance are you telling us about?
(Please **write in**)

____

Now answer the questions below:
(Please tick **one box per line**)

	Never/ almost never	Sometimes	Often	Always/ nearly always
Do you think your use was out of control?	☐ 0	☐ 1	☐ 2	☐ 3
Did the prospect of missing a fix (or dose) make you anxious or worried?	☐ 0	☐ 1	☐ 2	☐ 3
Did you worry about your use?	☐ 0	☐ 1	☐ 2	☐ 3
Did you wish you could stop?	☐ 0	☐ 1	☐ 2	☐ 3

	Not difficult	Quite difficult	Very difficult	Impossible
How difficult would you find it to stop or go without?	☐ 0	☐ 1	☐ 2	☐ 3

45

Listed below are a number of events.

Thinking about the last year, please read each item carefully and then answer in one of the following ways:

- **A No, the event has not happened**
- **B Yes, but I no longer feel affected by the event**
- **C Yes, and I am still affected by the event**

** immediate family includes: mother, father, sister, brother, partner, child*

(Please tick **one box per line**)

	A<br>No	B<br>Yes, but it does not affect me	C<br>Yes, and it still affects me
Have you had a serious illness or been seriously injured?	1	2	3
Has one of your immediate family* been seriously ill or injured?	1	2	3
Have any of your close friends or other close relatives been seriously ill or injured?	1	2	3
Have any of your immediate family died?	1	2	3
Have any of your other close relatives or close friends died?	1	2	3
Have you separated from your partner (not including death)?	1	2	3
Has a child living in your household been placed on the child protection register or been taken into care?	1	2	3
Have you had any serious problem with a close friend, neighbour or relative?	1	2	3
Have you, or an immediate family member been subject to serious racial abuse, attack or threats?	1	2	3
Have you, or an immediate family member been subject to any abuse, attack, threat – perhaps due to you or someone close to you having a disability of any kind (i.e. a mental health problem, a learning disability or a physical problem)?	1	2	3

	A<br>No	B<br>Yes, but it does not affect me	C<br>Yes, and it still affects me
Have you, or an immediate family member been subject to any other form of serious abuse, attack, or threat?	1	2	3
Have you or your partner been unemployed or seeking work for more than one month?	1	2	3
Have you or your partner been sacked from your job or made redundant?	1	2	3
Have you had any major financial difficulties (e.g. debts, difficulty paying bills)?	1	2	3
Have you, or an immediate family member had any police contact or been in a court appearance?	1	2	3
Have you or an immediate member of your family been burgled or mugged?	1	2	3
Has another individual who lives with you given birth?	1	2	3
Has another individual who lives with you suffered from a miscarriage or had a stillbirth?	1	2	3
Have you moved house (through choice)?	1	2	3
Have you moved house (not through choice)?	1	2	3
Have you had any housing difficulties?	1	2	3
Have you been homeless?	1	2	3
Have you had any other significant event happen? (Please **write in**)	1	2	3

46	**There are many areas of everyday life that can affect the health of you or your family members. The following questions are about any areas that make you feel worried or concerned.** (Please circle **one number per line**)

The physical and/or emotional health of my family causes me...

no concern	1	2	3	4	5	a lot of concern

My access to health and other services is...

good	1	2	3	4	5	poor

The support I have from family and friends is...

good	1	2	3	4	5	poor

Employment/unemployment within the family causes me...

no concern	1	2	3	4	5	a lot of concern

Money causes me...

no concern	1	2	3	4	5	a lot of concern

Housing causes me...

no concern	1	2	3	4	5	a lot of concern

The use of tobacco, alcohol or other drugs within the household causes me...

no concern	1	2	3	4	5	a lot of concern

Stressful life events, e.g. childhood experiences, abuse, domestic violence, crime etc., cause me...

no concern	1	2	3	4	5	a lot of concern

Being a parent causes me...

no concern	1	2	3	4	5	a lot of concern

Do you have any other worries not covered above? (Please **write in**)

47	**The following questions have been designed so that you can show how you have been feeling in the past week. Don't take too long over replies; your immediate reaction to each statement will probably be more accurate than a long, thought-out response.**<br><br>**Read each statement and tick the box that best describes you.**

I feel tense or 'wound up'
(Please tick **one box only**)

Most of the time	☐	3
A lot of the time	☐	2
From time to time, occasionally	☐	1
Not at all	☐	0

I still enjoy the things I used to enjoy
(Please tick **one box only**)

Definitely as much	☐	0
Not quite so much	☐	1
Only a little	☐	2
Hardly at all	☐	3

I get a sort of frightened feeling as if something awful is about to happen
(Please tick **one box only**)

Very definitely and quite badly	☐	3
Yes, but not too badly	☐	2
A little, but it doesn't worry me	☐	1
Not at all	☐	0

I can laugh and see the funny side of things
(Please tick **one box only**)

As much as I always could ☐ 0
Not quite so much now ☐ 1
Definitely not so much now ☐ 2
Not at all ☐ 3

Worrying thoughts go through my mind
(Please tick **one box only**)

A great deal of the time ☐ 3
A lot of the time ☐ 2
Not too often ☐ 1
Very little ☐ 0

I feel cheerful
(Please tick **one box only**)

Never ☐ 3
Not often ☐ 2
Sometimes ☐ 1
Most of the time ☐ 0

I can sit at ease and feel relaxed
(Please tick **one box only**)

Definitely ☐ 0
Usually ☐ 1
Not often ☐ 2
Not at all ☐ 3

I feel as if I am slowed down
(Please tick **one box only**)

- Nearly all the time ☐ 3
- Very often ☐ 2
- Sometimes ☐ 1
- Not at all ☐ 0

I get a sort of frightened feeling like 'butterflies' in the stomach
(Please tick **one box only**)

- Not at all ☐ 0
- Occasionally ☐ 1
- Quite often ☐ 2
- Very often ☐ 3

I have lost interest in my appearance
(Please tick **one box only**)

- Definitely ☐ 3
- I don't take as much care as I should ☐ 2
- I may not take quite as much care ☐ 1
- I take just as much care as ever ☐ 0

I feel restless as if I have to be on the move
(Please tick **one box only**)

- Very much indeed ☐ 3
- Quite a lot ☐ 2
- Not very much ☐ 1
- Not at all ☐ 0

I look forward with enjoyment to things
(Please tick **one box only**)

As much as I ever did ☐ 0

Rather less than I used to ☐ 1

Definitely less than I used to ☐ 2

Hardly at all ☐ 3

I get sudden feelings of panic
(Please tick **one box only**)

Very often indeed ☐ 3

Quite often ☐ 2

Not very often ☐ 1

Not at all ☐ 0

I can enjoy a good book or radio or television programme
(Please tick **one box only**)

Often ☐ 0

Sometimes ☐ 1

Not often ☐ 2

Very seldom ☐ 3

I lose my temper and shout and snap at others
(Please tick **one box only**)

Yes, definitely ☐ 3

Yes, sometimes ☐ 2

No, not much ☐ 1

No, not at all ☐ 0

I feel I might lose control and hit or hurt someone
(Please tick **one box only**)

Sometimes	☐	3
Occasionally	☐	2
Rarely	☐	1
Never	☐	0

I am patient with other people
(Please tick **one box only**)

All the time	☐	0
Most of the time	☐	1
Some of the time	☐	2
Hardly ever	☐	3

People upset me so that I feel like slamming doors or banging about
(Please tick **one box only**)

Yes, often	☐	3
Yes, sometimes	☐	2
Only occasionally	☐	1
Not at all	☐	0

48	Below is a list of problems people sometimes have. Please read each one carefully and <u>tick the box</u> that best describes how much that problem has distressed or bothered you <u>during the past 7 days</u>, including today. (Please tick **one box per line**)

**How much were you distressed by:**	**Not at all**	**A little bit**	**Moderately**	**Quite a bit**	**Extremely**
Nervousness or shakiness inside	0	1	2	3	4
Faintness or dizziness	0	1	2	3	4
The idea that someone else can control your thoughts	0	1	2	3	4
Feeling others are to blame for most of your troubles	0	1	2	3	4
Trouble remembering things	0	1	2	3	4
Feeling easily annoyed or irritated	0	1	2	3	4
Pains in heart or chest	0	1	2	3	4
Feeling afraid in open spaces or on the street	0	1	2	3	4
Thoughts of ending your life	0	1	2	3	4
Feeling that most people cannot be trusted	0	1	2	3	4
Poor appetite	0	1	2	3	4
Being suddenly scared for no reason	0	1	2	3	4
Temper outbursts that you could not control	0	1	2	3	4
Feeling lonely even when you are with people	0	1	2	3	4
Feeling blocked in getting things done	0	1	2	3	4
Feeling lonely	0	1	2	3	4
Feeling blue	0	1	2	3	4

How much were you distressed by:	Not at all	A little bit	Moderately	Quite a bit	Extremely
Feeling no interest in things	0	1	2	3	4
Feeling fearful	0	1	2	3	4
Your feelings being easily hurt	0	1	2	3	4
Feeling that people are unfriendly or dislike you	0	1	2	3	4
Feeling inferior to others	0	1	2	3	4
Nausea or upset stomach	0	1	2	3	4
Feeling that you are watched or talked about by others	0	1	2	3	4
Trouble falling asleep	0	1	2	3	4
Having to check and double-check what you do	0	1	2	3	4
Difficulty making decisions	0	1	2	3	4
Feeling afraid to travel on buses, subways or trains	0	1	2	3	4
Trouble getting your breath	0	1	2	3	4
Hot or cold spells	0	1	2	3	4
Avoiding certain things, places or activities because they frighten you	0	1	2	3	4
Your mind going blank	0	1	2	3	4
Numbness or tingling in parts of your body	0	1	2	3	4
The idea that you should be punished for your sins	0	1	2	3	4
Feeling hopeless about the future	0	1	2	3	4

How much were you distressed by:	Not at all	A little bit	Moderately	Quite a bit	Extremely
Trouble concentrating	0	1	2	3	4
Feeling weak in parts of your body	0	1	2	3	4
Feeling tense or keyed up	0	1	2	3	4
Thoughts of death or dying	0	1	2	3	4
Having urges to beat, injure or harm someone	0	1	2	3	4
Having urges to break or smash things	0	1	2	3	4
Feeling very self-conscious with others	0	1	2	3	4
Feeling uneasy in crowds, such as shopping or at a movie	0	1	2	3	4
Never feeling close to another person	0	1	2	3	4
Spells of terror or panic	0	1	2	3	4
Getting into frequent arguments	0	1	2	3	4
Feeling nervous when you are left alone	0	1	2	3	4
Others not giving you proper credit for your achievements	0	1	2	3	4
Feeling so restless you couldn't sit still	0	1	2	3	4
Feelings of worthlessness	0	1	2	3	4
Feeling that people will take advantage of you if you let them	0	1	2	3	4
Feelings of guilt	0	1	2	3	4
The idea that something is wrong with your mind	0	1	2	3	4

49	**By placing a tick in <u>one box in each group</u> below, please indicate which statements <u>best</u> describe your own health state today.**

Mobility

I have no problems in walking about ☐ 1

I have some problems in walking about ☐ 2

I am confined to bed ☐ 3

Self-Care

I have no problems with self-care ☐ 1

I have some problems washing or dressing myself ☐ 2

I am unable to wash or dress myself ☐ 3

Usual Activities (*e.g. work, study, housework, family or leisure activities*)

I have no problems with performing my usual activities ☐ 1

I have some problems with performing my usual activities ☐ 2

I am unable to perform my usual activities ☐ 3

Pain/Discomfort

I have no pain or discomfort ☐ 1

I have moderate pain or discomfort ☐ 2

I have extreme pain or discomfort ☐ 3

Anxiety/Depression

I am not anxious or depressed ☐ 1

I am moderately anxious or depressed ☐ 2

I am extremely anxious or depressed ☐ 3

THIS IS AN EXAMPLE

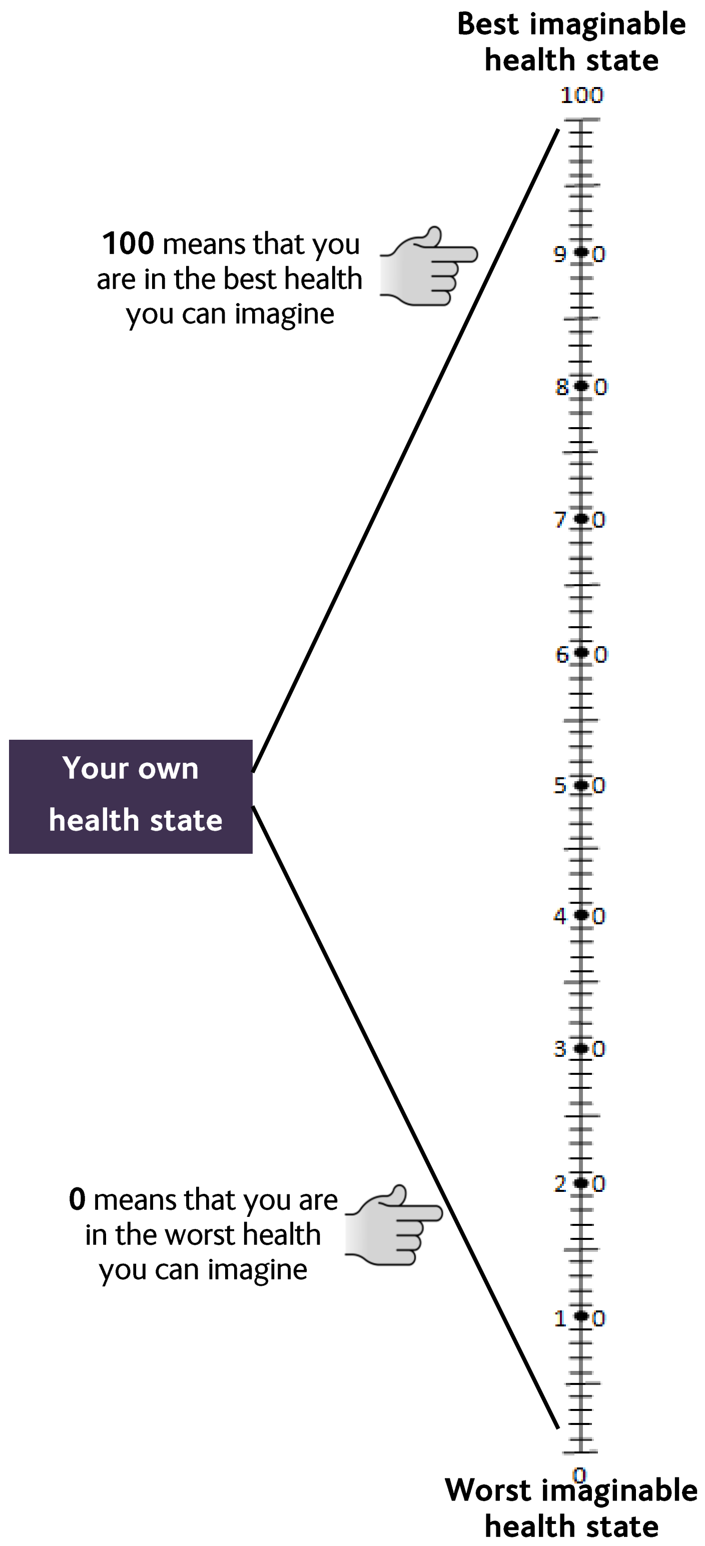

EQ-5D-3L

UK (English) © 1990 EuroQol Group EQ-5D™ is a trade mark of the EuroQol Gro

50 NOW IT IS YOUR TURN

To help people say how good or bad a health state is, we have drawn a scale (rather like a thermometer) on which the best state you can imagine is marked 100 and the worst state you can imagine is marked 0.

We would like you to indicate on this scale how good or bad your own health is today, in your opinion. Please do this by drawing a line from the box below to whichever point on the scale indicates how good or bad your health state is today.

Your own health state

Best imaginable health state

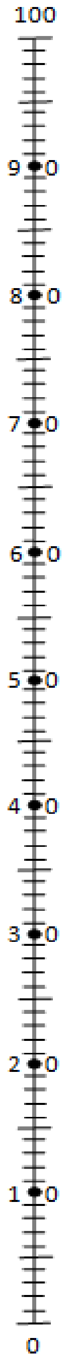

Worst imaginable health state

EQ-5D-3L

UK (English) © 1990 EuroQol Group EQ-5D™ is a trade mark of the EuroQol Gro

Your support

51	**During this pregnancy, who would help you if a problem came up?** (For example, who would help you if you needed to borrow £20 or if you got sick and had to be in bed for several weeks?) (Please tick **all that apply**)

- My husband or partner ☐ 1
- My mother, father, or in-laws ☐ 1
- Other family member or relative ☐ 1
- A friend ☐ 1
- Religious community (e.g. church, mosque) ☐ 1
- Someone else (please **write in**) ☐ 1

____

- No one would help me ☐ 1

52	**During this pregnancy, would you have the kinds of help listed below if you needed them?** (For each one, please **tick either Yes or No**)

	Yes	No
Someone to loan me £20	☐ 1	☐ 2
Someone to help me if I were sick and needed to be in bed	☐ 1	☐ 2
Someone to take me to the clinic or doctor's surgery if I needed a lift	☐ 1	☐ 2
Someone to talk with about my problems	☐ 1	☐ 2

Your partner

53 **Are you currently in a relationship?**
(Please tick **one box only**)

Yes ☐ 1 **Go to Question 54**

No ☐ 2 **Which of the following best describes you?** (Please tick **one box only**)

I am single/never married ☐ 3

I am divorced ☐ 4

I am separated ☐ 5

I am widowed ☐ 6

Now go to Question 71

54 **Which statement best describes you?**
(Please tick **one box only**)

I am married/in a civil partnership ☐ 1

I live with someone as a couple but we are not married/in a civil partnership ☐ 2

I am in a relationship but we do not live together as a couple ☐ 3

55 **How old is your partner?**
(Please **write in**, if you don't know, please write in your best guess)

______________________ Years old

56 **Is your partner male or female?**
(Please tick **one box only**)

Male ☐ 1

Female ☐ 2

57	**Is this your first pregnancy with your partner?** (Please tick **one box only**)

Yes ☐ 1

No ☐ 2

58	**Which ethnic background does your partner belong to?** (Please tick **all that apply**)

White

Scottish ☐ 1

British ☐ 1

English ☐ 1

Irish ☐ 1

Northern Irish ☐ 1

Welsh ☐ 1

Any other White background (Please **write in**) ☐ 1

____

I don't know their ethnic background ☐ 1

Asian or Asian British

Bangladeshi ☐ 1

Indian ☐ 1

Pakistani ☐ 1

Any other Asian background (Please **write in**) ☐ 1

____

Black or Black British

African ☐ 1

Caribbean ☐ 1

Any other Black background (Please **write in**) ☐ 1

____

59	**Which of these statements best describes your partner?** (Please tick **one box only**)

They are currently in paid employment or self-employed	☐	1	**Go to Question 60**
They are not currently working but have had paid employment in the past	☐	2	**Go to Question 61**
They have never been in paid employment	☐	3	**Go to Question 64**

60	**How many hours did they work last week?** (Please **write in**, if you don't know, please write in your best guess)

____________________ Hours

61	**Please tell us about their current job or their last job if they are not working.** (Please **write in**)

**Job title** Example: waiter	
**What this actually involves** Example: taking food orders, serving customers food and drink	
**Employer type** Example: restaurant	

62	**Which of these best describes their current job or their last job if they are not working now?** (Please tick **one box only**)

Self employed with paid employees	☐	1
Self employed with NO paid employees	☐	2
Manager	☐	3
Supervisor	☐	4
Employee	☐	5
Don't know	☐	6

63	**What size of company is/was it?** (Please tick **one box only**)

Under 25 staff ☐ 1

25 staff or more ☐ 2

Don't know ☐ 3

64	**Which describes best what your partner was doing last week?** (Please tick **one box only**)

In paid employment ☐ 1

Doing unpaid work for a business that they own or that a relative owns ☐ 2

Waiting to take up paid work already obtained ☐ 3

On a Government scheme for employment training ☐ 4

Looking for paid work on a Government training scheme ☐ 5

Intending to look for work but prevented by temporary sickness or illness ☐ 6

Permanently unable to work because of long-term sickness or disability ☐ 7

At college or university full time ☐ 8

At college or university part time ☐ 9

In full time secondary education (e.g. attending high school) ☐ 10

Retired from paid work ☐ 11

Looking after home or family ☐ 12

Providing full time care for an ill or disabled friend or relative ☐ 13

Doing something else (please **write in**) ☐ 14

____

____

65	Which of the following describes how your partner feels about the pregnancy?<br>(Please circle **all that apply**)

Protected	Unsure	Happy
Not bothered	Excited	Anxious
Resentful	Irritable	Relaxed
Loving	Worried	Supported
Angry	Confused	Calm
Don't care	Unhappy	Protective
Laid-back	Serious	Proud
Scared	Caring	Nervous
Strong	Other	Weak

↓

____

(Please **write in**)

66	How involved do you think your partner will be in your pregnancy and the baby's life?<br>(Please tick **one box per line**)

	Yes	Maybe	No
He/she will come to antenatal scans and appointments with me	☐ 1	☐ 2	☐ 3
He/she will come to antenatal classes with me	☐ 1	☐ 2	☐ 3
He/she will attend the birth	☐ 1	☐ 2	☐ 3
He/she will help me prepare for my baby's arrival	☐ 1	☐ 2	☐ 3
He/she will support my baby financially	☐ 1	☐ 2	☐ 3
He/she will be emotionally involved in my baby's life	☐ 1	☐ 2	☐ 3
He/she will see my baby as often as he/she can	☐ 1	☐ 2	☐ 3
He/she will be involved in my baby's upbringing	☐ 1	☐ 2	☐ 3

67	**I am unable to see or speak to my partner every day because...** (Please tick **one box per line**)

	Yes	No
he/she is currently living or working abroad	☐ 1	☐ 2
he/she is a serving member of the armed forces and deployed overseas	☐ 1	☐ 2
we do not live at the same address	☐ 1	☐ 2
I do not want to have contact with him/her	☐ 1	☐ 2
my family do not want me having contact with him/her	☐ 1	☐ 2
he/she is in prison	☐ 1	☐ 2
I have been told by a health or social care professional that if I have contact with him/her I will not be allowed to keep my baby	☐ 1	☐ 2
he/she was physically abusive towards me	☐ 1	☐ 2
other (please **write in**)	☐ 1	☐ 2

____

____

68	**The following questions are about how you have been feeling during the past month. If you have not thought about these issues during the past month, please answer the questions based on your present feelings.** (Please tick **one box per line**)

	Never	Rarely	Often	Very much
Has there been tension between you and your partner – irritability, unpleasant silence, etc?	☐ 1	☐ 2	☐ 3	☐ 4
Has your partner tried to share your interests?	☐ 1	☐ 2	☐ 3	☐ 4
Have you felt your partner went out too often without you?	☐ 1	☐ 2	☐ 3	☐ 4
Have you been feeling close to your partner since you became pregnant?	☐ 1	☐ 2	☐ 3	☐ 4
Does your partner show their approval of you?	☐ 1	☐ 2	☐ 3	☐ 4

	Very much	A lot	A little	Not at all
Has your partner helped in the running of the house?	☐ 4	☐ 3	☐ 2	☐ 1
Have you felt like putting your arms round your partner and cuddling him/her?	☐ 4	☐ 3	☐ 2	☐ 1
Do you enjoy spending time with your partner?	☐ 4	☐ 3	☐ 2	☐ 1

	Very often	Often	Rarely	Never
Have arguments between you and your partner come close to blows?	☐ 4	☐ 3	☐ 2	☐ 1
Have you found it easy to show affection to your partner?	☐ 4	☐ 3	☐ 2	☐ 1
Have you felt that your partner was paying you too little attention?	☐ 4	☐ 3	☐ 2	☐ 1
Has your partner seemed to ignore how you were feeling?	☐ 4	☐ 3	☐ 2	☐ 1
Has your partner shown affection to you?	☐ 4	☐ 3	☐ 2	☐ 1
Have you wished you could rely more on your partner to look after you?	☐ 4	☐ 3	☐ 2	☐ 1
Does your partner talk to you about his/her problems and feelings?	☐ 4	☐ 3	☐ 2	☐ 1

69	**Does your partner...** (Please tick **one box per line**)		
	**Yes**	**No**	**Don't know**
smoke cigarettes/cigars?	☐ 1	☐ 2	☐ 3
smoke cannabis?	☐ 1	☐ 2	☐ 3
use illegal (street) drugs? e.g. heroin, crack/cocaine, valium, ecstasy	☐ 1	☐ 2	☐ 3
inject illegal (street) drugs? e.g. heroin, crack/cocaine, temazepam	☐ 1	☐ 2	☐ 3
take prescribed opiate substitute drugs? e.g. methadone or buprenorphine (Subutex/Suboxone)	☐ 1	☐ 2	☐ 3
receive help from services for his/her alcohol use?	☐ 1	☐ 2	☐ 3
receive help from services for his/her drug use?	☐ 1	☐ 2	☐ 3

70 Is your partner the father of your unborn child?
(Please tick **one box only**)

Yes ☐ 1 **Go to Question 85**

No ☐ 2 **Go to Question 71**

Not sure ☐ 3 **Go to Question 85**

The father of your baby

71	**Have you told the father of your unborn child that you are pregnant?** (Please tick **one box only**)

Yes ☐ 1

No ☐ 2 **Go to Question 85**

I'm not sure who the father is ☐ **Go to Question 85**

72	**Is this your first pregnancy with the father of your unborn child?** (Please tick **one box only**)

Yes ☐ 1

No ☐ 2

73	**How old is the father of your unborn child?** (Please **write in**, if you don't know, please write in your best guess)

________________ Years old

74	**Which of the following describes how the father of your baby feels about the pregnancy?** (Please circle **all that apply**)

Protected	Unsure	Happy
Not bothered	Excited	Anxious
Resentful	Irritable	Relaxed
Loving	Worried	Supported
Angry	Confused	Calm
Don't care	Unhappy	Protective
Laid-back	Serious	Proud
Scared	Caring	Nervous
Strong	Other ↓	Weak

____

(Please **write in**)

75	**How often do you usually see or speak to the father of your unborn child?** (Please tick **one box only**)

- Not at all ☐ 1
- Less than once a month ☐ 2
- 1 – 2 times a month ☐ 3
- About once a week ☐ 4
- Most days ☐ 5
- Every day ☐ 6

76	**I am unable to see or speak to the father of my baby every day because...** (Please tick **one box per line**)

	Yes	No
he is currently living or working abroad	☐ 1	☐ 2
he is a serving member of the armed forces and deployed overseas	☐ 1	☐ 2
we do not live at the same address	☐ 1	☐ 2
I do not want to have contact with him	☐ 1	☐ 2
my family do not want me having contact with him	☐ 1	☐ 2
he is in prison	☐ 1	☐ 2
I have been told by a health or social care professional that if I have contact with him I will not be allowed to keep my baby	☐ 1	☐ 2
he was physically abusive towards me	☐ 1	☐ 2
other (please **write in**)	☐ 1	☐ 2

____

____

77	**How involved do you think the father of your unborn child will be in your baby's life?**<br>(Please tick **one box per line**)

	Yes	Maybe	No
He will come to antenatal scans and appointments with me	☐ 1	☐ 2	☐ 3
He will come to antenatal classes with me	☐ 1	☐ 2	☐ 3
He will attend the birth	☐ 1	☐ 2	☐ 3
He will help me prepare for my baby's arrival	☐ 1	☐ 2	☐ 3
He will support my baby financially	☐ 1	☐ 2	☐ 3
He will be emotionally involved in my baby's life	☐ 1	☐ 2	☐ 3
He will see my baby as often as he can	☐ 1	☐ 2	☐ 3
He will be involved in my baby's upbringing	☐ 1	☐ 2	☐ 3

78	**Does the father of your baby...**<br>(Please tick **one box per line**)

	Yes	No	Don't know
smoke cigarettes/cigars?	☐ 1	☐ 2	☐ 3
smoke cannabis?	☐ 1	☐ 2	☐ 3
use illegal (street) drugs? e.g. heroin, crack/cocaine, valium, ecstasy	☐ 1	☐ 2	☐ 3
inject illegal (street) drugs? e.g. heroin, crack/cocaine, temazepam	☐ 1	☐ 2	☐ 3
take prescribed opiate substitute drugs? e.g. methadone or buprenorphine (Subutex/Suboxone)	☐ 1	☐ 2	☐ 3
receive help from services for his alcohol use?	☐ 1	☐ 2	☐ 3
receive help from services for his drug use?	☐ 1	☐ 2	☐ 3

79	**Which of these statements best describes the father of your baby?** (Please tick **one box only**)

They are currently in paid employment or self-employed	☐ 1	**Go to Question 80**
They are not currently working but have had paid employment in the past	☐ 2	**Go to Question 81**
They have never been in paid employment	☐ 3	**Go to Question 84**

80	**How many hours did they work last week?** (Please **write in**, if you don't know, please write in your best guess)

_______________ Hours

81	**Please tell us about their current job or their last job if they are not working.** (Please **write in**)

**Job title**<br>Example: waiter	
**What this actually involves**<br>Example: taking food orders, serving customers food and drink	
**Employer type**<br>Example: restaurant	

82	**Which of these best describes their current job or their last job if they are not working now?** (Please tick **one box only**)

Self employed with paid employees	☐ 1
Self employed with NO paid employees	☐ 2
Manager	☐ 3
Supervisor	☐ 4
Employee	☐ 5
Don't know	☐ 6

83	**What size of company is/was it?** (Please tick **one box only**)

Under 25 staff ☐ 1

25 staff or more ☐ 2

Don't know ☐ 3

84	**Which describes best what the father of your baby was doing last week?** (Please tick **one box only**)

In paid employment ☐ 1

Doing unpaid work for a business that he owns or that a relative owns ☐ 2

Waiting to take up paid work already obtained ☐ 3

On a Government scheme for employment training ☐ 4

Looking for paid work on a Government training scheme ☐ 5

Intending to look for work but prevented by temporary sickness or illness ☐ 6

Permanently unable to work because of long-term sickness or disability ☐ 7

At college or university full time ☐ 8

At college or university part time ☐ 9

In full time secondary education (e.g. attending high school) ☐ 10

Retired from paid work ☐ 11

Looking after home or family ☐ 12

Providing full time care for an ill or disabled friend or relative ☐ 13

Doing something else (please **write in**) ☐ 14

____

____

Your childhood

Pregnancy is often a time when women reflect upon their own childhood and their relationship with their parents. We would now like to ask you some questions about what your childhood was like.

85 Looking back would you say that your childhood was happy?
(Please tick **one box per line**)

When you were	Very happy	Quite happy	Not really happy	Quite unhappy	Very unhappy	Can't remember
0 – 5 years old	☐ 1	☐ 2	☐ 3	☐ 4	☐ 5	☐ 6
6 – 11 years old	☐ 1	☐ 2	☐ 3	☐ 4	☐ 5	☐ 6
12 – 15 years old	☐ 1	☐ 2	☐ 3	☐ 4	☐ 5	☐ 6

86 Did your parents divorce or separate before your 18th birthday?
(Please tick **one box only**)

Yes ☐ 1 **Go to Question 86**

No ☐ 2 **Go to Question 88**

87 How old were you when your parents divorced or separated?
(Please **write in**)

_______________ Years old

88 Who did you mainly live with after this?
(Please tick **one box only**)

Mum ☐ 1 Dad ☐ 2 Sometimes Mum, sometimes Dad ☐ 3

Someone else (please **write in**) ☐ 4

____

89	**When I was growing up...**<br>(Please tick **one box per line**)

	**Never true**	**Rarely true**	**Sometimes true**	**Often true**	**Very often true**
I didn't have enough to eat	1	2	3	4	5
I knew that there was someone to take care of me and protect me	1	2	3	4	5
people in my family called me things like 'stupid', 'lazy' or 'ugly'	1	2	3	4	5
my parents were too drunk or high to take care of the family	1	2	3	4	5
there was someone in my family who helped me feel that I was important or special	1	2	3	4	5
I had to wear dirty clothes	1	2	3	4	5
I felt loved	1	2	3	4	5
I thought that my parents wished I had never been born	1	2	3	4	5
I got hit so hard by someone in my family that I had to see a doctor or go to the hospital	1	2	3	4	5
there was nothing I wanted to change about my family	1	2	3	4	5
people in my family hit me so hard that it left me with bruises or marks	1	2	3	4	5
I was punished with a belt, a board, a cord or some other hard object	1	2	3	4	5
people in my family looked out for each other	1	2	3	4	5
people in my family said hurtful or insulting things to me	1	2	3	4	5

	Never true	Rarely true	Sometimes true	Often true	Very often true
I believe that I was physically abused	1	2	3	4	5
I had the perfect childhood	1	2	3	4	5
I got hit or beaten so badly that it was noticed by someone like a teacher, neighbour or doctor	1	2	3	4	5
I felt that someone in my family hated me	1	2	3	4	5
people in my family felt close to each other	1	2	3	4	5
someone tried to touch me in a sexual way or tried to make me touch them	1	2	3	4	5
someone threatened to hurt me or tell lies about me unless I did something sexual with them	1	2	3	4	5
I had the best family in the world	1	2	3	4	5
someone tried to make me do sexual things or watch sexual things	1	2	3	4	5
someone molested me	1	2	3	4	5
I believe that I was emotionally abused	1	2	3	4	5
there was someone to take me to the doctor if I needed it	1	2	3	4	5
I believe that I was sexually abused	1	2	3	4	5
my family was a source of strength and support	1	2	3	4	5

90	**Did any of the following happen to you during childhood?** (Please tick **one box per line**)

	Yes	No	Not sure
I was legally adopted	1	2	3
I lived in a children's home or residential unit/school	1	2	3
I lived with a foster carer	1	2	3
I lived in secure accommodation or a young person's institute/prison	1	2	3
I lived with a relative (other than for holidays or short visits)	1	2	3

Parenting

91	**The following questions are about things that sometimes cause problems for parents in the first few months after their baby is born. How well do you think you would cope with the problems listed in the first three months after your baby is born?** (Please tick **one box per line**)

	Very well	Quite well	Not well	Not well at all	Does not apply
Managing the relationship between my baby and his/her brothers or sisters	1	2	3	4	5
My baby suffering from wind or colic	1	2	3	4	5
My baby's sleeping pattern	1	2	3	4	5
Getting my baby to feed	1	2	3	4	5
My baby having health problems	1	2	3	4	5
Being able to afford all the baby clothes and equipment you need for your baby	1	2	3	4	5
Managing the house and other domestic responsibilities (e.g. cooking, cleaning, shopping)	1	2	3	4	5

92	Please say how much you agree or disagree with each statement. (Please tick **one box per line**)				
	**Strongly agree**	**Agree**	**Neither agree nor disagree**	**Disagree**	**Strongly disagree**
Nobody can teach you how to be a good parent, you just have to learn for yourself	☐ 1	☐ 2	☐ 3	☐ 4	☐ 5
If you ask for help or advice on parenting from professionals like doctors or social workers they will start interfering or trying to take over	☐ 1	☐ 2	☐ 3	☐ 4	☐ 5
It's difficult to ask people for help or advice about parenting unless you know them really well	☐ 1	☐ 2	☐ 3	☐ 4	☐ 5
It's hard to know who to ask for help or advice about being a parent	☐ 1	☐ 2	☐ 3	☐ 4	☐ 5

93 **How much experience do you have spending time with or looking after very young children?**
(Please tick **one box only**)

**A lot**	**Quite a lot**	**Not very much**	**None at all**
☐ 1	☐ 2	☐ 3	☐ 4

END OF QUESTIONNAIRE

THANK YOU VERY MUCH FOR TAKING PART AND ANSWERING THE QUESTIONS

**If you have any thoughts on this questionnaire or feedback for the THRIVE team that may help us inform future aspects of this research, please feel free to leave comments here. You can also email your comments to THRIVE@sphsu.mrc.ac.uk**
