# Supplementary material for: Trial of healthy relationship initiatives for the very early years (THRIVE), evaluating Enhanced Triple P for Baby and Mellow Bumps additional social and care needs during pregnancy and their infants who are at higher risk of maltreatment: study protocol for a randomised controlled trial
Source: Trials. 2019 Aug 14;20:499. doi: 10.1186/s13063-019-3571-5 (PMC6694522; doi:10.1186/s13063-019-3571-5)
Supplement: Supplementary file 3 — THRIVE follow-up questionnaire (v2.1 25.01.16). Questionnaire completed by study participants at the follow-up visit (6 months postnatal). (PDF 2561 kb) [file 13063_2019_3571_MOESM3_ESM.pdf]

University of Glasgow

Medical Research Council

MRC

CHIEF SCIENTIST OFFICE

Trial of Healthy Relationship Initiatives for the Very Early-years

Follow up 1

Questionnaire

NHS Ayrshire & Arran

NHS Greater Glasgow and Clyde

For administration purposes only

Date: ☐ ☐ / ☐ ☐ / ☐ ☐ ☐ ☐

Participant ID: ____________________

Researcher ID: ____________________

Circle as appropriate: **SC / SCHR / RC**

Your answers will remain confidential and will not be seen by:

- **your family or friends**
- **your health or social care practitioner**

So please be honest about how you feel and what you think.

There are no 'right' or 'wrong' answers. We just want to know what you think and something about your experiences.

Please read the instructions carefully.

If you are not sure what a question means please ask the researcher.

If you do not want to answer a question, please just leave it blank and go on to the next question.

Your pregnancy and giving birth to your baby

The following questions are about your experiences of being pregnant with your six month old son or daughter.

1 During pregnancy, women are offered a number of appointments with midwives and doctors at community and hospital-based antenatal clinics. Which of the following statements best describes how many antenatal appointments you attended?

(Please tick **one box only**)

I attended all of my appointments	☐	1
I attended most of my appointments	☐	2
I attended some of my appointments	☐	3

2 Which of these statements best describes your attendance at NHS antenatal classes during your pregnancy with your 6 month old baby?

(Please tick **one box only**)

I went to all of my antenatal classes	☐	1	**Go to Question 4**
I went to most of my antenatal classes	☐	2	**Go to Question 3**
I went to some of my antenatal classes	☐	3	**Go to Question 3**
I did not attend antenatal classes	☐	4	**Go to Question 3**
I was not offered NHS antenatal classes	☐	5	**Go to Question 7**

The THRIVE Trial is funded by the National Institute for Health Research's Public Health Research Programme

3	**Why did you not attend all of the NHS antenatal classes?** (Please tick **all that apply**)
	I didn't like the classes so I stopped going ☐
	I did not want to go to classes ☐
	I attended classes for a previous pregnancy ☐
	There was nothing I needed/wanted to know ☐
	The location of the class was not easy for me to travel to ☐
	I couldn't get childcare while at the class ☐
	It cost too much to travel to the class ☐
	I do not like classes/groups ☐
	I went to private classes not run by the NHS ☐
	There were no classes available/accessible ☐
	I was too embarrassed/shy to go ☐
	I could not get the time off work ☐
	My partner did not want me to go ☐
	I felt the group I went to as part of this study gave me all the information I needed ☐
	I thought I was not able to go because I went to a group as part of this study ☐
	I was not well enough to go to classes ☐
	Other reasons (Please **write in** below) ☐
	______________________________

The THRIVE Trial is funded by the National Institute for Health Research's Public Health Research Programme

4	**Did your partner/the father of your baby go to any of the NHS antenatal classes?** (Please tick **one box only**)

No, because I did not attend classes	☐ 1	**Go to Question 7**
Yes, they went to most or all of the classes	☐ 2	**Go to Question 7**
Yes, they went to some of the classes	☐ 3	**Go to Question 7**
No, they did not attend any classes	☐ 4	**Go to Question 5**

5	**Did anyone else go to the NHS antenatal classes with you instead of your partner/father of your baby?** (Please tick **all that apply**)

No, I went alone	☐ 1
Yes, my mum	☐ 1
Yes, another family member	☐ 1
Yes, my friend	☐ 1
Yes, my key worker	☐ 1
Yes, someone else (Please **write in** below)	☐ 1

____

The THRIVE Trial is funded by the National Institute for Health Research's Public Health Research Programme

6	**Why did your partner/the father of your baby not go with you to the NHS antenatal classes?** (Please tick **all that apply**)

- They did not want to go to classes ☐
- They had attended classes for a previous pregnancy ☐
- There was nothing they needed/wanted to know ☐
- The location of the class was not easy for them to travel to ☐
- We couldn't get childcare ☐
- It would have cost too much for them to go to the class ☐
- They do not like classes/groups ☐
- I did not want them to come with me ☐
- I am not in contact with them ☐
- They could not get the time off work ☐
- They didn't want to have anything to do with my baby ☐
- Partners were not invited to attend ☐
- I don't know why they did not attend ☐
- Other (Please **write in** below) ☐

____

The THRIVE Trial is funded by the National Institute for Health Research's Public Health Research Programme

7 **Have you attended any of the following during your pregnancy or since your baby has been born?**
(Please tick **one box per line**)

	Yes, I went alone	Yes, I went with my partner/ father of my baby	Yes, I went with someone else	No
Private antenatal classes (e.g. NCT, Lazy Daisy)	1	2	3	4
Mellow Bumps (not run by the THRIVE Trial)	1	2	3	4
Mellow Mums, Mellow Babies or Mellow Toddlers	1	2	3	4
Triple P for Baby (not run by the THRIVE Trial)	1	2	3	4
A Triple P seminar or group (not Triple P for Baby)	1	2	3	4
Minding the Baby	1	2	3	4
Young Parent Support Groups	1	2	3	4
Barnardo's Threads	1	2	3	4
Family Nurse Practitioner	1	2	3	4
Homestart	1	2	3	4
Family Support Groups	1	2	3	4
Baby Massage	1	2	3	4
Other (Please **write in** below)	1	2	3	4

____

The THRIVE Trial is funded by the National Institute for Health Research's Public Health Research Programme

The following questions are about your experiences of giving birth to your six month old son or daughter.

8 **Please complete the table for your 6 month old baby or babies.**
(Please fill out **one line for each baby** you gave birth to)

Child	Date of birth	Sex: M	Sex: F	Was this child born before 37 weeks gestation? Yes	Was this child born before 37 weeks gestation? No	What was the birth weight of your baby? (Please **write in,** either pounds **or** grams) Lbs	oz	OR	grams
1	DD / MM / YY	1	2	1	2				
2	DD / MM / YY	1	2	1	2				
3	DD / MM / YY	1	2	1	2				

9 **Did your baby spend time in a special care baby unit or neo-natal intensive care unit after they were born?**
(Please tick **one box only**)

Yes ☐ 1 **Go to Question 10**

No ☐ 2 **Go to Question 11**

10 **Approximately how long did they spend in the special care baby unit or neo-natal intensive care unit after they were born?**
(Please **write in**)

Baby 1 ☐ **Weeks** ☐ **Days**

Baby 2 ☐ **Weeks** ☐ **Days**

Baby 3 ☐ **Weeks** ☐ **Days**

The THRIVE Trial is funded by the National Institute for Health Research's Public Health Research Programme

11 Where did you give birth?
(Please tick **one box only**)

At home	☐	1
In a midwifery led unit	☐	2
In a consultant led unit at a hospital	☐	3
I gave birth somewhere else (e.g. in an ambulance)	☐	4
Other (Please **write in** below)	☐	5

____

12 Which of the following best describes how you gave birth?
(Please tick **one box only**)

I had a vaginal birth	☐	1
I had a forceps delivery	☐	2
I had a ventouse/kiwi assisted delivery	☐	3
I had a caesarean section prior to labour beginning (it was planned)	☐	4
I had a caesarean section after labour began (it was not planned)	☐	5
I don't know	☐	6

The THRIVE Trial is funded by the National Institute for Health Research's Public Health Research Programme

13	**Did any of the following stop your birth from going the way you had planned?** (Please tick **all that apply**)
	No, my birth went as I had planned ☐
	I needed to have my baby by caesarean section ☐
	I needed more pain relief than I had planned ☐
	My blood pressure went up ☐
	I bled before I had my baby ☐
	I was induced (my labour was started for me) ☐
	My baby was breech (bottom first) ☐
	My birthing partner missed the birth ☐
	My baby was distressed and the doctors were worried ☐
	My baby passed meconium (baby's first black/green poo) in labour ☐
	I needed forceps ☐
	Other (Please **write in** below) ☐
	____________________

14 **This question asks about your experience of the labour and birth of your baby. If you had a planned caesarean, please answer the questions as best you can for how your experience was. If a question is not relevant to your experience, please tick 'neither'.**

What kind of control did you have during labour and birth? (Please tick one box per line)

	Agree completely	Agree slightly	Neither	Disagree slightly	Disagree completely
I had control over when procedures happened	5	4	2	3	1
I could influence which procedures were carried out	5	4	2	3	1
I decided whether most procedures were carried out or not	5	4	2	3	1
I had control over the decisions that were made	5	4	2	3	1
The people in the room took control	5	4	2	3	1
People coming in and out of the room was beyond my control	5	4	2	3	1
I could get up and move around as much as I wanted	5	4	2	3	1
I chose whether I was given information or not	5	4	2	3	1
I could decide when I received information	5	4	2	3	1
I had control over what information I was given	5	4	2	3	1
I felt I had control over the way my baby was finally born	5	4	2	3	1
The pain was too great for me to gain control over it	5	4	2	3	1
I was overcome by the pain	5	4	2	3	1
I was mentally calm	5	4	2	3	1
I was able to control my reactions to the pain	5	4	2	3	1
I was in control of my emotions	5	4	3	2	1

The THRIVE Trial is funded by the National Institute for Health Research's Public Health Research Programme

	Agree completely	Agree slightly	Neither	Disagree slightly	Disagree completely
I felt my body was on a mission that I could not control	5	4	3	2	1
Negative feelings overwhelmed me	5	4	3	2	1
I gained control by working with my body	5	4	3	2	1
I behaved in a way not like myself	5	4	3	2	1
I could control the sounds I was making	5	4	3	2	1
The staff helped me find energy to continue when I wanted to give up	5	4	3	2	1
The staff knew instinctively what I wanted or needed	5	4	3	2	1
The staff went out of their way to try to keep me comfortable	5	4	3	2	1
The staff encouraged me to try new ways of coping	5	4	3	2	1
The staff encouraged me not to fight against what my body was doing	5	4	3	2	1
The staff realised the pain I was in	5	4	3	2	1
I felt the staff had their own agenda	5	4	3	2	1
I was given time to ask questions	5	4	3	2	1
I felt like the staff tried to move things along for their own convenience	5	4	3	2	1
The staff helped me to try different positions	5	4	3	2	1
The staff stopped doing something if I asked them to stop	5	4	3	2	1
The staff dismissed things I said to them	5	4	3	2	1

The THRIVE Trial is funded by the National Institute for Health Research's Public Health Research Programme

15 After giving birth to your baby did any of the following things happen? (Please tick all that apply)

I experienced complications that required medication (e.g. my blood pressure went up)	☐	1
I had to have emergency surgery after my baby was born	☐	1
I developed an infection	☐	1
I experienced complications that resulted in a longer than planned stay in hospital	☐	1
I was admitted to an intensive care unit	☐	1
I was readmitted to hospital after I took my baby home (e.g. I had to go back into hospital)	☐	1
I was admitted to a mother and baby unit	☐	1
My baby was kept in hospital after he/she was born for medical treatment and I was discharged home	☐	1
I had a postpartum haemorrhage (e.g. I bled too much)	☐	1
My baby was readmitted to hospital after I took them home (e.g. my baby had to go back into hospital)	☐	1
My baby was removed by social work services	☐	1
Other (Please **write in** below)	☐	1

____

16 Other than health professionals; did you have anybody with you when you gave birth?
(Please tick **one box only**)

Yes	☐	1	**Go to Question 17**
No	☐	2	**Go to Question 18**

17 Please complete the table below to tell us who was with you when you gave birth and how supportive you felt they were. An example of how to complete the table is provided.

Please fill out **one line for each person** who was with you when you gave birth.
Do not include health professionals.

Their relationship to you	How supportive were they? Very supportive	Supportive	Neither supportive or unsupportive	Unsupportive	Very unsupportive
**Example –** My partner	1	✓ 2	3	4	5
	1	2	3	4	5
	1	2	3	4	5
	1	2	3	4	5
	1	2	3	4	5
	1	2	3	4	5

The THRIVE Trial is funded by the National Institute for Health Research's Public Health Research Programme

Some families require additional support. The following questions are about the level of support you received from Social Work while you were pregnant.

18	**Did your midwife refer you to social work whilst you were pregnant?** (Please tick **one box only**)		
	Yes	☐ 1	**Go to Question 19**
	No	☐ 2	**Go to Question 22**

19	**What were the reasons that you were referred to social work?** (Please tick **all that apply**)	
	I had a history of misusing drugs and/or alcohol	☐ 1
	My partner had a history of misusing drugs and/or alcohol	☐ 1
	I had been involved with the criminal justice system	☐ 1
	My partner had been involved with the criminal justice system	☐ 1
	There were concerns about my parenting abilities	☐ 1
	There were concerns about my partner's/the father of my baby's parenting abilities	☐ 1
	My partner/the father of my baby was abusive towards me	☐ 1
	I had previously been involved with social work	☐ 1
	My partner had previously been involved with social work	☐ 1
	I have had previous children placed into care	☐ 1
	My partner/the father of my baby has had previous children placed into care	☐ 1
	I have a history of severe mental health difficulties	☐ 1
	My partner/the father of my baby has a history of severe mental health difficulties	☐ 1
	I had been in the care system	☐ 1
	My partner/the father of my baby had been in the care system	☐ 1
	Other reasons (Please **write in** below)	☐ 1
	______________________________	

The THRIVE Trial is funded by the National Institute for Health Research's Public Health Research Programme

20	**Was a pre-birth case conference held for your baby?** (Please tick **one box only**)

Yes ☐ 1 **Go to Question 21**

No ☐ 2 **Go to Question 22**

21	**Which of the following statements apply to you?** **(Please tick all that apply)**

I attended the pre-birth case conference ☐ 1

The father of my baby attended the pre-birth case conference ☐ 1

My partner (if different from the father of your baby) attended the pre-birth case conference ☐ 1

Your health and wellbeing

22	**Are you pregnant?** (Please tick **one box only**)

Yes ☐ 1

No ☐ 2

23	**Which of these statements best describes you?** (Please tick **one box only**)

Smoker ☐ 1 **Go to Question 25**

Ex-smoker ☐ 2 **Go to Question 24**

Non-smoker ☐ 3 **Go to Question 26**

24	**How long has it been since you stopped smoking?** (Please **write in**, if you're not sure then please write in your best guess)

________ years ________ months ________ days

The THRIVE Trial is funded by the National Institute for Health Research's Public Health Research Programme

25 On average how many cigarettes did you smoke per day ...
(Please tick **one box per column**)

	during your pregnancy?	in the last seven days?
None	☐ 1	☐ 1
10 or less	☐ 2	☐ 2
11 – 20	☐ 3	☐ 3
21 – 30	☐ 4	☐ 4
More than 30	☐ 5	☐ 5

26 How often did you drink until you felt drunk?
(Please tick **one box per column**)

	during your pregnancy?	since your baby was born?
Every day per week	☐ 1	☐ 1
A few days per week	☐ 2	☐ 2
A few days per month	☐ 3	☐ 3
Once or more per month	☐ 4	☐ 4
Never	☐ 5	☐ 5

27 Have you ever smoked cannabis? (e.g. skunk, weed, hash)
(Please tick **all that apply**)

No ☐ 1

Yes, in the past ☐ 1

Yes, during my pregnancy ☐ 1

Yes, in the last 6 months ☐ 1

The THRIVE Trial is funded by the National Institute for Health Research's Public Health Research Programme

28	**Have you ever taken any other illegal (street) drug? (e.g. heroin, crack/cocaine, non-prescribed valium, ecstasy)** (Please tick **all that apply**)

No	☐ 1	**Go to Question 32**
Yes, in the past	☐ 1	**Go to Question 29**
Yes, during my pregnancy	☐ 1	**Go to Question 29**
Yes, in the last 6 months	☐ 1	**Go to Question 29**

29	**Have you ever injected any illegal (street) drug? (e.g. heroin, crack/cocaine, non-prescribed temazepam, amphetamines)** (Please tick **all that apply**)

No	☐ 1	Yes, in the past	☐ 1
Yes, during my pregnancy	☐ 1	Yes, in the last 6 months	☐ 1

30	**Are you currently being prescribed an opiate substitute drug? (e.g. methadone or buprenorphine (Subutex/Suboxone))** (Please tick **one box only**)

No	☐ 1	**Go to Question 32**
Methadone	☐ 2	**Go to Question 31**
Subutex/Suboxone	☐ 3	**Go to Question 31**

31	**Are you using opiate substitute drugs that are not prescribed to you? (e.g. street methadone or buprenorphine (Subutex/Suboxone))** (Please tick **one box only**)

No	☐ 1
Yes, I am buying it from someone	☐ 2
Yes, I have been prescribed an opiate substitute **and** I am also buying it from someone	☐ 3

The THRIVE Trial is funded by the National Institute for Health Research's Public Health Research Programme

32 **Have you been diagnosed with any of the following conditions or illnesses?**
(Please tick **all that apply**)

	**before pregnancy?**	**during pregnancy?**	**after pregnancy?**
Asthma	☐ 1	☐ 1	☐ 1
Diabetes	☐ 1	☐ 1	☐ 1
Depression	☐ 1	☐ 1	☐ 1
High blood pressure	☐ 1	☐ 1	☐ 1
Anxiety	☐ 1	☐ 1	☐ 1
Hyper or Hypothyroidism	☐ 1	☐ 1	☐ 1
Bipolar	☐ 1	☐ 1	☐ 1
DVT or Embolism (Blood Clots)	☐ 1	☐ 1	☐ 1
A Sexually Transmitted Infection	☐ 1	☐ 1	☐ 1
Epilepsy	☐ 1	☐ 1	☐ 1
Other (please **write in** below)	☐ 1	☐ 1	☐ 1

____

If you have never been diagnosed with any of these conditions, please **tick this box** ☐ 1

33 **Do you think that you experienced mental ill health (e.g. depression, stress or anxiety)?**
(Please tick **one box per column**)

	**before your pregnancy?**	**during your pregnancy?**	**in the three months after your baby was born?**	**between four and six months after your baby was born?**
Yes	☐ 1	☐ 1	☐ 1	☐ 1
No	☐ 2	☐ 2	☐ 2	☐ 2
Don't know	☐ 3	☐ 3	☐ 3	☐ 3

The THRIVE Trial is funded by the National Institute for Health Research's Public Health Research Programme

34	**Have you ever been referred to and met with a...** (Please tick **all that apply**)		
	**before your pregnancy?**	**during your pregnancy?**	**since your baby was born?**
Community Psychiatric Nurse	☐ 1	☐ 1	☐ 1
Mental Health Support Worker (i.e. SAMH, GAMH)	☐ 1	☐ 1	☐ 1
Mental Health Nurse (not community based)	☐ 1	☐ 1	☐ 1
Counsellor/Therapist	☐ 1	☐ 1	☐ 1
Addiction Support Worker	☐ 1	☐ 1	☐ 1

If you have never been referred or met with any of the above mental health professionals please **tick this box** ☐ 1

35 **The following questions have been designed so that you can show how you have been feeling in the past week.**
Don't take too long over replies; your immediate reaction to each statement will probably be more accurate than a long, thought-out response.
Read each statement and tick the box that best describes you.

I feel tense or 'wound up' (Please tick **one box only**)

- Most of the time ☐ 3
- A lot of the time ☐ 2
- From time to time, occasionally ☐ 1
- Not at all ☐ 0

I still enjoy the things I used to enjoy (Please tick **one box only**)

- Definitely as much ☐ 0
- Not quite so much ☐ 1
- Only a little ☐ 2
- Hardly at all ☐ 3

**I get a sort of frightened feeling as if something awful is about to happen** (Please tick **one box only**)	
Very definitely and quite badly	☐ 3
Yes, but not too badly	☐ 2
A little, but it doesn't worry me	☐ 1
Not at all	☐ 0

**I can laugh and see the funny side of things** (Please tick **one box only**)	
As much as I always could	☐ 0
Not quite so much now	☐ 1
Definitely not so much now	☐ 2
Not at all	☐ 3

**Worrying thoughts go through my mind** (Please tick **one box only**)	
A great deal of the time	☐ 3
A lot of the time	☐ 2
Not too often	☐ 1
Very little	☐ 0

**I feel cheerful** (Please tick **one box only**)	
Never	☐ 3
Not often	☐ 2
Sometimes	☐ 1
Most of the time	☐ 0

The THRIVE Trial is funded by the National Institute for Health Research's Public Health Research Programme

**I can sit at ease and feel relaxed** (Please tick **one box only**)		
Definitely	☐	0
Usually	☐	1
Not often	☐	2
Not at all	☐	3

**I feel as if I am slowed down** (Please tick **one box only**)		
Nearly all the time	☐	3
Very often	☐	2
Sometimes	☐	1
Not at all	☐	0

**I get a sort of frightened feeling like 'butterflies' in the stomach** (Please tick **one box only**)		
Not at all	☐	0
Occasionally	☐	1
Quite often	☐	2
Very often	☐	3

**I have lost interest in my appearance** (Please tick **one box only**)		
Definitely	☐	3
I don't take as much care as I should	☐	2
I may not take quite as much care	☐	1
I take just as much care as ever	☐	0

The THRIVE Trial is funded by the National Institute for Health Research's Public Health Research Programme

**I feel restless as if I have to be on the move** (Please tick **one box only**)		
Very much indeed	☐	3
Quite a lot	☐	2
Not very much	☐	1
Not at all	☐	0

**I look forward with enjoyment to things** (Please tick **one box only**)		
As much as I ever did	☐	0
Rather less than I used to	☐	1
Definitely less than I used to	☐	2
Hardly at all	☐	3

**I get sudden feelings of panic** (Please tick **one box only**)		
Very often indeed	☐	3
Quite often	☐	2
Not very often	☐	1
Not at all	☐	0

**I can enjoy a good book or radio or television programme** (Please tick **one box only**)		
Often	☐	0
Sometimes	☐	1
Not often	☐	2
Very seldom	☐	3

The THRIVE Trial is funded by the National Institute for Health Research's Public Health Research Programme

**I lose my temper and shout and snap at others** (Please tick **one box only**)		
Yes, definitely	☐	3
Yes, sometimes	☐	2
No, not much	☐	1
No, not at all	☐	0

**I feel I might lose control and hit or hurt someone** (Please tick **one box only**)		
Sometimes	☐	3
Occasionally	☐	2
Rarely	☐	1
Never	☐	0

**I am patient with other people** (Please tick **one box only**)		
All the time	☐	0
Most of the time	☐	1
Some of the time	☐	2
Hardly ever	☐	3

**People upset me so that I feel like slamming doors or banging about** (Please tick **one box only**)		
Yes, often	☐	3
Yes, sometimes	☐	2
Only occasionally	☐	1
Not at all	☐	0

The THRIVE Trial is funded by the National Institute for Health Research's Public Health Research Programme

36	**By placing a tick in <u>one box in each group</u> below, please indicate which statements <u>best</u> describe your own health state today.**

Mobility

I have no problems in walking about ☐ 1

I have some problems in walking about ☐ 2

I am confined to bed ☐ 3

Self-Care

I have no problems with self-care ☐ 1

I have some problems washing or dressing myself ☐ 2

I am unable to wash or dress myself ☐ 3

Usual Activities (*e.g. work, study, housework, family or leisure activities*)

I have no problems with performing my usual activities ☐ 1

I have some problems with performing my usual activities ☐ 2

I am unable to perform my usual activities ☐ 3

Pain/Discomfort

I have no pain or discomfort ☐ 1

I have moderate pain or discomfort ☐ 2

I have extreme pain or discomfort ☐ 3

Anxiety/Depression

I am not anxious or depressed ☐ 1

I am moderately anxious or depressed ☐ 2

I am extremely anxious or depressed ☐ 3

37	**To help people say how good or bad a health state is, we have drawn a scale (rather like a thermometer) on which the best state you can imagine is marked 100 and the worst state you can imagine is marked 0.**

We would like you to indicate on this scale how good or bad your own health is today, in your opinion. Please do this by drawing a line from the box below to whichever point on the scale indicates how good or bad your health state is today.

Best imaginable

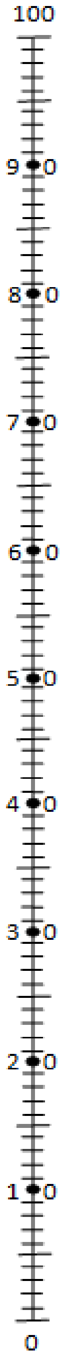

Worst imaginable health state

Your own health state

EQ-5D-3L

Ul [illegible] Qol Group EQ-5D™ is a trade mark of the EuroQol Gro

The THRIVE Trial is funded by the National Institute for Health Research's Public Health Research Programme

38	**Has your ability to care for or interact with your baby been affected by any health problem or disability?** (Please tick **one box only**)

Yes ☐ 1 **Go to Question 39**

No ☐ 2 **Go to Question 41**

39	**What is your health condition/disability?** (Please **write in**)

____

40	**How much does your health problem/disability affect your ability to ...** (Please tick **one box per line**)

	**A lot**	**A little**	**Not at all**
feed your baby?	☐ 1	☐ 2	☐ 3
pick up your baby?	☐ 1	☐ 2	☐ 3
change nappies?	☐ 1	☐ 2	☐ 3
dress your baby?	☐ 1	☐ 2	☐ 3
carry your baby?	☐ 1	☐ 2	☐ 3
hold your baby?	☐ 1	☐ 2	☐ 3
cuddle with your baby?	☐ 1	☐ 2	☐ 3
speak to your baby?	☐ 1	☐ 2	☐ 3
read to your baby?	☐ 1	☐ 2	☐ 3
hear/listen to your baby?	☐ 1	☐ 2	☐ 3
bath your baby?	☐ 1	☐ 2	☐ 3
play with your baby?	☐ 1	☐ 2	☐ 3
go outside with your baby or take your baby places?	☐ 1	☐ 2	☐ 3
be happy with your baby?	☐ 1	☐ 2	☐ 3
Other (please **write in**)	☐ 1	☐ 2	☐ 3

____

The THRIVE Trial is funded by the National Institute for Health Research's Public Health Research Programme

41 **Listed below are a number of events.**
Thinking about the last year, please read each item carefully and then answer in one of the following ways:

A **No, the event has not happened**
B **Yes, but I no longer feel affected by the event**
C **Yes, and I am still affected by the event**

** immediate family includes: mother, father, sister, brother, partner, child*

(Please tick **one box per line**)	**A** No	**B** Yes, but it does not affect me	**C** Yes, and it still affects me
Have you had a serious illness or been seriously injured?	☐ 1	☐ 2	☐ 3
Has one of your immediate family* been seriously ill or injured?	☐ 1	☐ 2	☐ 3
Have any of your close friends or other close relatives been seriously ill or injured?	☐ 1	☐ 2	☐ 3
Have any of your immediate family died?	☐ 1	☐ 2	☐ 3
Have any of your other close relatives or close friends died?	☐ 1	☐ 2	☐ 3
Have you separated from your partner (not including death)?	☐ 1	☐ 2	☐ 3
Has a child living in your household been placed on the child protection register or been taken into care?	☐ 1	☐ 2	☐ 3
Have you had any serious problem with a close friend, neighbour or relative?	☐ 1	☐ 2	☐ 3
Have you, or an immediate family member been subject to serious racial abuse, attack or threats?	☐ 1	☐ 2	☐ 3
Have you, or an immediate family member been subject to any abuse, attack, threat – perhaps due to you or someone close to you having a disability of any kind (i.e. a mental health problem, a learning disability or a physical problem)?	☐ 1	☐ 2	☐ 3

The THRIVE Trial is funded by the National Institute for Health Research's Public Health Research Programme

(Please tick **one box per line**)	**A** No	**B** Yes, but it does not affect me	**C** Yes, and it still affects me
Have you or an immediate family member been subject to any other form of serious abuse, attack or threat?	1	2	3
Have you or your partner been unemployed or seeking work for more than one month?	1	2	3
Have you or your partner been sacked from your job or made redundant?	1	2	3
Have you had any major financial difficulties (e.g. debts, difficulty paying bills)?	1	2	3
Have you, or an immediate family member had any police contact or been in a court appearance?	1	2	3
Have you or an immediate member of your family been burgled or mugged?	1	2	3
Has another individual who lives with you given birth?	1	2	3
Has another individual who lives with you suffered from a miscarriage or had a stillbirth?	1	2	3
Have you moved house (through choice)?	1	2	3
Have you moved house (not through choice)?	1	2	3
Have you had any housing difficulties?	1	2	3
Have you been homeless?	1	2	3
Have you had any other significant event happen? (Please **write in** below)			
______________________	1	2	3
______________________	1	2	3

The THRIVE Trial is funded by the National Institute for Health Research's Public Health Research Programme

42 How do you cope with events?

Everyone gets confronted with negative or unpleasant events now and then and everyone responds to them in his or her own way. By the following questions you are asked to indicate what you generally think, when you experience negative or unpleasant events.
(Please tick **one box per line**)

	(Almost) never	Sometimes	Regularly	Often	(Almost) always
I think that I have to accept that this has happened	1	2	3	4	5
I often think about how I feel about what I have experienced	1	2	3	4	5
I think I can learn something from the situation	1	2	3	4	5
I feel that I am the one who is responsible for what has happened	1	2	3	4	5
I think that I have to accept the situation	1	2	3	4	5
I am preoccupied with what I think and feel about what I have experienced	1	2	3	4	5
I think of pleasant things that have nothing to do with it	1	2	3	4	5
I think that I can become a stronger person as a result of what has happened	1	2	3	4	5
I keep thinking about how terrible it is what I have experienced	1	2	3	4	5

The THRIVE Trial is funded by the National Institute for Health Research's Public Health Research Programme

	(Almost) never	Sometimes	Regularly	Often	(Almost) always
I feel that others are responsible for what has happened	1	2	3	4	5
I think of something nice instead of what has happened	1	2	3	4	5
I think about how to change that situation	1	2	3	4	5
I think that it hasn't been too bad compared to other things	1	2	3	4	5
I think that basically the cause must lie within myself	1	2	3	4	5
I think about a plan of what I can do best	1	2	3	4	5
I tell myself that there are worse things in life	1	2	3	4	5
I continually think how horrible the situation has been	1	2	3	4	5
I feel that basically the cause lies with others	1	2	3	4	5

The THRIVE Trial is funded by the National Institute for Health Research's Public Health Research Programme

Your baby's health and development

43 **In general, how is your baby's health?**
(Please tick one box only)

- Very good ☐ 1
- Good ☐ 2
- Fair ☐ 3
- Bad ☐ 4
- Very bad ☐ 5

44 **Which of the following options best describes how you have fed your baby?**
(Please tick **all that apply**)

	Before 6 weeks	Between 6–17 weeks	Between 17–26 weeks
I breastfed my baby	☐ 1	☐ 1	☐ 1
I fed my baby formula milk	☐ 1	☐ 1	☐ 1
I gave my baby solids (e.g. baby rice)	☐ 1	☐ 1	☐ 1

45 **In general, which do you believe about breast or bottle during the first three months?**
(Please tick **one box only**)

- Babies should be fed whenever and for as long as they want ☐ 1
- Babies should be allowed unrestricted sucking, including night feeds, but the idea of "meal times" should be introduced ☐ 2
- Babies should be fed whenever they are clearly hungry ☐ 3
- Babies should be fed adjustable quantities at specified times but not at night ☐ 4
- Babies should be fed a set amount by schedule (e.g. 3-4 hourly with no 'snacking' in between) ☐ 5

The THRIVE Trial is funded by the National Institute for Health Research's Public Health Research Programme

46	**Ideally, when should weaning occur?**<br>(Please **write in your answer in months**)
	Babies should be ___________ months old

47	**When do you believe that babies begin trying to communicate?**<br>**(Please tick one box only)**
	During pregnancy/before birth ☐ 1
	At birth ☐ 2
	Within the first two weeks of birth ☐ 3
	Between 3 to 8 weeks after being born ☐ 4
	When they are aged 2 months old or more ☐ 5

48	**How confident do you feel to help your child's speech and language develop?**<br>(Please tick **one box only**)
	Not sure ☐ 1
	Not confident ☐ 2
	A bit confident ☐ 3
	Very confident ☐ 4

The THRIVE Trial is funded by the National Institute for Health Research's Public Health Research Programme

49	**How confident do you feel that you know what developmental stage your child should be for their age?** (Please tick **one box only**)

Not sure	☐	1
Not much confidence	☐	2
A bit confident	☐	3
Very confident	☐	4

50	**At what age do you think most children start to say their first words? The words may not be clear yet.** (Please tick **one box only**)

6 – 12 months	☐	1
13 – 18 months	☐	2
19 – 24 months	☐	3

51	**Has any member of your baby's family*...** * by family we mean both your and the father of your baby's family (Please tick **one box per line**)

	Yes	No	Don't know
been late to talk?	☐ 1	☐ 2	☐ 3
had ongoing problems with speech/language during childhood?	☐ 1	☐ 2	☐ 3
had problems with stuttering?	☐ 1	☐ 2	☐ 3
had problems learning to read?	☐ 1	☐ 2	☐ 3

The THRIVE Trial is funded by the National Institute for Health Research's Public Health Research Programme

52

Only complete this question if you said 'yes' to a statement in Question 51

Please specify/give details of the problem that was experienced. Examples are provided to help you.

**Example 1:**	My brother had to go to see a speech and language therapist when he was a child because he had trouble saying some words.
**Example 2:**	My baby's father and his mother can't read or write.
**Example 3:**	I am dyslexic.

(Please **write in**)

53

Does your baby have any health problems or disabilities that are expected to be long term?

(Please tick **one box only**)

Yes ☐ 1 **Please give more details in the space below**

No ☐ 2

The THRIVE Trial is funded by the National Institute for Health Research's Public Health Research Programme

54 Here is a list of things that some babies are able to do or learning to do by the time they are 6 months old. Please don't worry if your baby cannot do all of these things yet as your baby will develop at his/her own pace.

Does your baby...

(Please tick **one box per line**)	**Yes**	**No**	**Tries to do**
laugh, chuckle and squeal aloud in play or screams when annoyed?	☐ 1	☐ 2	☐ 3
push up with arms when on his or her stomach?	☐ 1	☐ 2	☐ 3
vocalise tunefully to self and others using sing-song vowel sounds or single or double syllables (e.g.a-a, ga-ga, adah muh) ?	☐ 1	☐ 2	☐ 3
smile?	☐ 1	☐ 2	☐ 3
turn immediately to familiar voices across the room?	☐ 1	☐ 2	☐ 3
sit up by themselves?	☐ 1	☐ 2	☐ 3
be happy with familiar people?	☐ 1	☐ 2	☐ 3
sometimes show anxiety about strangers when approached too nearly or abruptly, especially if a familiar adults is out of sight?	☐ 1	☐ 2	☐ 3
watch your face very closely?	☐ 1	☐ 2	☐ 3
reach and grab for objects/toys?	☐ 1	☐ 2	☐ 3
enjoy turn taking games such as peek-a-boo?	☐ 1	☐ 2	☐ 3
roll over?	☐ 1	☐ 2	☐ 3
hold his/her head up without support?	☐ 1	☐ 2	☐ 3
point at interesting things and show that they are excited by them?	☐ 1	☐ 2	☐ 3
hold and shake an object?	☐ 1	☐ 2	☐ 3
turn to the source when he/she hears sounds at ear level?	☐ 1	☐ 2	☐ 3
show interest in interacting with you?	☐ 1	☐ 2	☐ 3
listen to adult voices if not in view?	☐ 1	☐ 2	☐ 3
show a delighted response to active play?	☐ 1	☐ 2	☐ 3
bang a toy?	☐ 1	☐ 2	☐ 3

55	How often do you (and/or your partner) do the following with your baby? (Please tick **one box per line**)							
	**Every day/ most days**	**Once or twice a week**	**Once a fortnight**	**Once every 1–2 months**	**Once every 3–4 months**	**Once every 6 months**	**Once a year or less often**	**Never**
Go to the library	☐ 1	☐ 2	☐ 3	☐ 4	☐ 5	☐ 6	☐ 7	☐ 8
Read books or tell stories	☐ 1	☐ 2	☐ 3	☐ 4	☐ 5	☐ 6	☐ 7	☐ 8
Recite nursery rhymes or sing songs	☐ 1	☐ 2	☐ 3	☐ 4	☐ 5	☐ 6	☐ 7	☐ 8
Messy play (e.g. painting)	☐ 1	☐ 2	☐ 3	☐ 4	☐ 5	☐ 6	☐ 7	☐ 8
Soft play	☐ 1	☐ 2	☐ 3	☐ 4	☐ 5	☐ 6	☐ 7	☐ 8
Mother and toddler groups	☐ 1	☐ 2	☐ 3	☐ 4	☐ 5	☐ 6	☐ 7	☐ 8
Visit parks/ play parks	☐ 1	☐ 2	☐ 3	☐ 4	☐ 5	☐ 6	☐ 7	☐ 8
Go swimming	☐ 1	☐ 2	☐ 3	☐ 4	☐ 5	☐ 6	☐ 7	☐ 8
Go to nursery	☐ 1	☐ 2	☐ 3	☐ 4	☐ 5	☐ 6	☐ 7	☐ 8
Visit friends and family	☐ 1	☐ 2	☐ 3	☐ 4	☐ 5	☐ 6	☐ 7	☐ 8
Meet and play with friends or family who have young children	☐ 1	☐ 2	☐ 3	☐ 4	☐ 5	☐ 6	☐ 7	☐ 8

The THRIVE Trial is funded by the National Institute for Health Research's Public Health Research Programme

56	**About how many children's books do you have in your home at the moment, <u>including library books</u>, that are aimed at children under the age of 5?** (Please **circle one box**)

None [1]	1 – 5 [2]	6 – 10 [3]	11 – 20 [4]	20 + [5]

57	**About how many children's audio tapes or CDs do you have in your home at the moment, <u>including those borrowed from the library</u>, that are aimed at children under the age of 5?** (Please **circle one box**)

None [1]	1 – 5 [2]	6 – 10 [3]	11 – 20 [4]	20 + [5]

58	**About how many children's videos or DVDs do you have in your home at the moment, <u>including those borrowed from the library</u>, that are aimed at children under the age of 5?** (Please **circle one box**)

None [1]	1 – 5 [2]	6 – 10 [3]	11 – 20 [4]	20 + [5]

Relationships and support

59	**Are you currently in a relationship?** (Please tick **one box only**)

Yes ☐ 1 **Go to Question 60**

No ☐ 2 **Which of the following best describes you?**

(Please tick **one box only**)

I am single/never married ☐ 3

I am divorced ☐ 4

I am separated ☐ 5

I am widowed ☐ 6

Now go to Question 73

The THRIVE Trial is funded by the National Institute for Health Research's Public Health Research Programme

60 **Which statement best describes you?**

(Please tick **one box only**)

I am married/in a civil partnership ☐ 1

I live with someone as a couple but we are not married/ in a civil partnership ☐ 2

I am in a relationship but we do not live together as a couple ☐ 3

61 **Is this the <u>same</u> person that you were in a relationship with when we interviewed you previously?**

(Please tick **one box only**)

Yes	☐ 1	**Go to Question 65**
No	☐ 2	**Go to Question 62**
Don't know	☐ 3	**Go to Question 62**

62 **What sex is your partner?**

(Please tick **one box only**)

Male ☐ 1

Female ☐ 2

63 **How old is your partner?**

(Please **write in**, if you don't know, please write in your 'best guess')

__________ Years old

The THRIVE Trial is funded by the National Institute for Health Research's Public Health Research Programme

64 Which ethnic background does your partner belong to?

(Please tick **all that apply**)

White

- Scottish ☐ 1
- British ☐ 1
- English ☐ 1
- Irish ☐ 1
- Northern Irish ☐ 1
- Welsh ☐ 1
- Any other White background (Please **write in**) ☐ 1

____

I don't know their ethnic background ☐ 1

Asian or Asian British

- Bangladeshi ☐ 1
- Indian ☐ 1
- Pakistani ☐ 1
- Any other Asian background (Please **write in**) ☐ 1

____

Black or Black British

- African ☐ 1
- Caribbean ☐ 1
- Any other Black background (Please **write in**) ☐ 1

____

65 Which statements best describes your partner?

(Please tick **one box only**)

- They are currently in paid employment or self-employed ☐ 1
- They are not currently working but have had paid employment in the past ☐ 2
- They have never been in paid employment ☐ 3

66	**Does your partner...** (Please tick **one box per line**)		
	**Yes**	**No**	**Don't know**
smoke cigarettes/cigars?	1	2	3
smoke cannabis?	1	2	3
use illegal (street) drugs? e.g. heroin, crack/cocaine, non-prescribed valium	1	2	3
inject illegal (street) drugs? e.g. heroin, crack/cocaine, non-prescribed temazepam	1	2	3
take prescribed opiate substitute drugs? e.g. methadone or buprenorphine (Subutex/Suboxone)	1	2	3
receive help from services for their alcohol use?	1	2	3
receive help from services for their drug use?	1	2	3

67	**Did any of the following happen to your partner during childhood?** (Please tick **one box per line**)		
	**Yes**	**No**	**Not sure**
They were legally adopted	1	2	3
They lived in a children's home or residential unit/school	1	2	3
They lived with a foster carer	1	2	3
They lived in secure accommodation or a young person's institute/prison	1	2	3
They lived with a relative (other than for holidays or short visits)	1	2	3

68 The following questions are about the role that your partner plays in caring for the household and your baby. Please read each statement and circle the number that best describes how often you feel your partner does these things.

(Please circle **one number per line**)

How often...	Almost never	←				→	Very often
does your partner help take care of the baby (feeding, changing, bathing?)	1	2	3	4	5	6	7
do they play with the baby?	1	2	3	4	5	6	7
do they soothe the baby when he/she is upset?	1	2	3	4	5	6	7
do they watch the baby so you can go out by yourself?	1	2	3	4	5	6	7
do they help in other household chores?	1	2	3	4	5	6	7
do they help out with the family meals?	1	2	3	4	5	6	7
do they help with grocery shopping?	1	2	3	4	5	6	7
do they disagree with you about how the baby should be handled?	1	2	3	4	5	6	7
do they indicate to you by words or behaviour that they knows that it is hard work to take care of a baby?	1	2	3	4	5	6	7
do they indicate dissatisfaction with the change in routine since the baby's birth?	1	2	3	4	5	6	7
do they indicate dissatisfaction with the amount of time you have to spend together since the baby's birth?	1	2	3	4	5	6	7
do you talk about the baby with your partner?	1	2	3	4	5	6	7
do you confide in, share you problems with, or tell your troubles to your partner?	1	2	3	4	5	6	7
does your partner confide in, share their problems with, or tell you their troubles?	1	2	3	4	5	6	7
In general, do you feel your partner has been supportive since the baby's birth?	1	2	3	4	5	6	7
do they take your baby on outings or day trips?	1	2	3	4	5	6	7
do they buy essential items such as food, milk and clothing for the baby?	1	2	3	4	5	6	7
do they buy presents and toys for the baby?	1	2	3	4	5	6	7

The THRIVE Trial is funded by the National Institute for Health Research's Public Health Research Programme

69 How often do you do the following things to encourage your partner to be involved in child care and with your baby, including feeding, play, and emotional support?

(Please circle **one number per line**)

	Never ←				→	Several times a day
Tell your partner to do a child care task ("Go wash Jack's face.")	1	2	3	4	5	6
Ask your partner politely to help ("Can you wash Sophie's face please?")	1	2	3	4	5	6
Compliment your partner ("You're able to calm Jack down better than I can.")	1	2	3	4	5	6
Invite your partner to help ("Wouldn't you like to read to Sophie?")	1	2	3	4	5	6
Refuse to do it yourself ("I'm not giving Jack a bath, it's your turn.")	1	2	3	4	5	6
Give your partner a serious look that means, "You need to deal with Sophie now!"	1	2	3	4	5	6
Let your partner know you appreciate their contributions ("It really helps when you take Jack with you.")	1	2	3	4	5	6
Give your partner an irritated or exasperated look.	1	2	3	4	5	6
Hint that work needs to be done	1	2	3	4	5	6
Wait until your partner does child care tasks on their own	1	2	3	4	5	6
Leave the house so your partner doesn't have a choice	1	2	3	4	5	6
Ask your partner for help by "talking through" the baby ("Daddy help me, I've got a stinky nappy!")	1	2	3	4	5	6
Tell your partner what a good parent they are	1	2	3	4	5	6
Ask for your partner's opinion ("Do you think Jack should wear a jumper today?")	1	2	3	4	5	6
Tell other people about what a good parent they are at a time when they can hear you	1	2	3	4	5	6
Tell your partner how happy they make your baby ("Sophie really loves to play with you.")	1	2	3	4	5	6
Encourage your partner to spend time alone with your baby	1	2	3	4	5	6
Arrange activities for your partner and child to do together	1	2	3	4	5	6

The THRIVE Trial is funded by the National Institute for Health Research's Public Health Research Programme

70 **Having a new baby can sometimes affect the relationship that you have with your partner. The following questions are about how you have been feeling during the past month.**
If you have not thought about these issues during the past month, please answer the questions based on your present feelings.
(Please tick **one box per line**)

	Never	Rarely	Often	Very much
Has there been tension between you and your partner – irritability, unpleasant silence, etc?	1	2	3	4
Has your partner tried to share your interests?	1	2	3	4
Have you felt your partner went out too often without you?	1	2	3	4
Have you been feeling close to your partner since your baby was born?	1	2	3	4

	Very much	A lot	A little	Not at all
Has your partner helped in the running of the house?	4	3	2	1
Have you felt like putting your arms round your partner and cuddling him/her?	4	3	2	1

	Very often	Often	Rarely	Never
Have arguments between you and your partner come close to blows?	1	2	3	4
Have you found it easy to show affection to your partner?	1	2	3	4
Have you felt that your partner was paying you too little attention?	1	2	3	4
Has your partner seemed to ignore how you were feeling?	1	2	3	4
Has your partner shown affection to you?	1	2	3	4
Have you wished you could rely more on your partner to look after you?	1	2	3	4

The THRIVE Trial is funded by the National Institute for Health Research's Public Health Research Programme

71	Do you feel that having a baby has… (Please tick **one box only**)	
	brought you and your partner closer together	☐ 1
	made you feel less close than before	☐ 2
	made no difference to your relationship	☐ 3
	none of the above as we only started our relationship after my baby was born	☐ 4

72	**Is your current partner your baby's biological father?** (Please tick **one box only**)	
	Yes ☐ 1	**Go to Question 80**
	No ☐ 2	**Go to Question 73**

73	**Does your baby currently have any contact with his/her biological father?** (Please tick **one box only**)	
	Yes ☐ 1	**Go to Question 74**
	No ☐ 2	**Go to Question 75**

74 The following questions are about your baby's relationship with his/her biological father. How often does …

(Please tick **one box per line**)

	**Every day**	**5–6 times a week**	**3–4 times a week**	**1–2 times a week**	**At least once a month**	**Less than once a month**
your baby see his/her biological father?	☐ 1	☐ 2	☐ 3	☐ 4	☐ 5	☐ 6
your baby stay overnight with his/her biological father?	☐ 1	☐ 2	☐ 3	☐ 4	☐ 5	☐ 6
your baby's biological father take him/her on outings or day trips?	☐ 1	☐ 2	☐ 3	☐ 4	☐ 5	☐ 6
your baby's biological father buy toys or clothes for the baby, excluding birthdays and other special occasions?	☐ 1	☐ 2	☐ 3	☐ 4	☐ 5	☐ 6

The THRIVE Trial is funded by the National Institute for Health Research's Public Health Research Programme

75 Do any of the following prevent your baby from having contact with his/her biological father?

(Please tick **one box per line**)

	Yes	No
He is currently living or working abroad	1	2
He is a serving member of the armed forces and deployed overseas	1	2
We do not live at the same address	1	2
I do not want to have contact with him	1	2
My family do not want me having contact with him	1	2
He is in prison	1	2
I have been told by a health or social care professional that if I have contact with him I will not be allowed to keep my baby	1	2
He was physically abusive towards me or my baby	1	2
There is a child protection order that limits his contact with my baby	1	2
He died	1	2
Other (please **write in**)	1	2

76 How would you describe your relationship with your baby's biological father?

(Please tick **one box only**)

- Very friendly 1
- Friendly 2
- Neither friendly nor unfriendly 3
- Unfriendly 4
- Very unfriendly 5
- I do not see my baby's biological father 6

77	**Which of these statements <u>best</u> describes the father of your baby?** (Please tick **one box only**)	
	He is currently in paid employment or self-employed	☐ 1
	He is not currently working but has had paid employment in the past	☐ 2
	He has never been in paid employment	☐ 3
	I don't know	☐ 4

78	**Does the father of your baby…** (Please tick **one box per line**)	**Yes**	**No**	**Don't know**
	smoke cigarettes/cigars?	☐ 1	☐ 2	☐ 3
	smoke cannabis?	☐ 1	☐ 2	☐ 3
	use illegal (street) drugs? e.g. heroin, crack/cocaine, non-prescribed valium	☐ 1	☐ 2	☐ 3
	inject illegal (street) drugs? e.g. heroin, crack/cocaine, non-prescribed temazepam	☐ 1	☐ 2	☐ 3
	take prescribed opiate substitute drugs? e.g. methadone or buprenorphine (Subutex/Suboxone)	☐ 1	☐ 2	☐ 3
	receive help from services for their alcohol use?	☐ 1	☐ 2	☐ 3
	receive help from services for their drug use?	☐ 1	☐ 2	☐ 3

The THRIVE Trial is funded by the National Institute for Health Research's Public Health Research Programme

79 **Did any of the following happen to the father of your baby during his childhood?**
(Please tick **one box per line**)

	Yes	No	Not sure
He was legally adopted	☐ 1	☐ 2	☐ 3
He lived in a children's home or residential unit/school	☐ 1	☐ 2	☐ 3
He lived with a foster carer	☐ 1	☐ 2	☐ 3
He lived in secure accommodation or a young person's institute/prison	☐ 1	☐ 2	☐ 3
Helived with a relative (other than for holidays or short visits)	☐ 1	☐ 2	☐ 3

80 **Does your baby live with you every day of the week?**
(Please tick **one box only**)

Yes ☐ 1 **Go to Question 83**

No ☐ 2 **Go to Question 81**

81 **Who does your baby live with when they are not living with you?**
(Please **write in**)

____

82 **How many days a week does your baby usually stay with that person?**
(Please **circle**)

1 2 3 4 5 6 7

The THRIVE Trial is funded by the National Institute for Health Research's Public Health Research Programme

83	**Do any of the following people help you care for your baby?** (Please tick **all that apply**)

Nobody, I care for my baby by myself	☐	1
My partner	☐	1
My baby's father (if different from your partner)	☐	1
My mum	☐	1
My dad	☐	1
Another relative	☐	1
A friend	☐	1
Other (please **write in**, e.g. key worker, social worker)	☐	1

____

84	**Since your baby was born, have you had the kinds of help listed below if you needed them?** (Please tick **one box per line**)

	Yes	No
Someone to loan me £20	☐ 1	☐ 2
Someone to help me if I were sick and needed to be in bed	☐ 1	☐ 2
Someone to take me to the clinic or doctor's surgery if I needed a lift	☐ 1	☐ 2
Someone to talk to about my problems	☐ 1	☐ 2

85	**Who would help you if a problem came up?** (For example, who would help you if you needed to borrow £20 or if you got sick and had to be in bed for several weeks.)<br>(Please tick **all that apply**)

- My husband or partner ☐
- My mother, father or in-laws ☐
- Other family member or relative ☐
- A friend ☐
- Religious community (e.g. church, mosque) ☐
- Someone else (please **write in**) ☐

____

- No one would help me ☐

The THRIVE Trial is funded by the National Institute for Health Research's Public Health Research Programme

86 **How easy has it been for you to get help or advice from the following people since your baby was born?**
(Please tick **one box per line**)

	Very easy	Easy	Unsure	Not easy	Not easy at all	I haven't done this
My family doctor/GP	☐ 1	☐ 2	☐ 3	☐ 4	☐ 5	☐ 6
My health visitor	☐ 1	☐ 2	☐ 3	☐ 4	☐ 5	☐ 6
My social worker	☐ 1	☐ 2	☐ 3	☐ 4	☐ 5	☐ 6
Other mothers with small children	☐ 1	☐ 2	☐ 3	☐ 4	☐ 5	☐ 6
Drop-in centre for families	☐ 1	☐ 2	☐ 3	☐ 4	☐ 5	☐ 6
Telephone advice line	☐ 1	☐ 2	☐ 3	☐ 4	☐ 5	☐ 6
My family	☐ 1	☐ 2	☐ 3	☐ 4	☐ 5	☐ 6
The father of my baby	☐ 1	☐ 2	☐ 3	☐ 4	☐ 5	☐ 6
My partner (if different from father of baby)	☐ 1	☐ 2	☐ 3	☐ 4	☐ 5	☐ 6

87	**We are interested in how you feel about the following statements. Read each statement carefully. Indicate how you feel about each statement.** (Please **circle one number per line**)						
	**Very Strongly Disagree**	**Strongly Disagree**	**Mildly Disagree**	**Neutral**	**Mildly Agree**	**Strongly Agree**	**Very Strongly Agree**
There is a special person who is around when I am in need.	1	2	3	4	5	6	7
There is a special person that I can share my joys and sorrows with.	1	2	3	4	5	6	7
My family really tries to help me.	1	2	3	4	5	6	7
I get the emotional help and support I need from my family.	1	2	3	4	5	6	7
I have a special person who is a real source of comfort to me.	1	2	3	4	5	6	7
My friends really try to help me.	1	2	3	4	5	6	7
I can count on my friends when things go wrong.	1	2	3	4	5	6	7
I can talk about my problems with my family.	1	2	3	4	5	6	7
I have friends that I can share my joys and sorrows with.	1	2	3	4	5	6	7
There is a special person in my life who cares about my feelings.	1	2	3	4	5	6	7
My family is willing to help me make decisions.	1	2	3	4	5	6	7
I can talk about my problems with my friends.	1	2	3	4	5	6	7

The THRIVE Trial is funded by the National Institute for Health Research's Public Health Research Programme

Parenting and motherhood

88 **The following questions are about how you care for your baby.**
Please tick the answer that comes closest to how you generally feel. (Please tick one box per line)

	No, hardly ever	No, not very often	Yes, some of the time	Yes, most of the time	N/A
I am confident about feeding my baby	☐ 0	☐ 1	☐ 2	☐ 3	☐ 4
I can settle my baby	☐ 0	☐ 1	☐ 2	☐ 3	☐ 4
I am confident about helping my baby to establish a good sleeping routine	☐ 0	☐ 1	☐ 2	☐ 3	☐ 4
I know what to do when my baby cries	☐ 0	☐ 1	☐ 2	☐ 3	☐ 4
I understand what my baby is trying to tell me	☐ 0	☐ 1	☐ 2	☐ 3	☐ 4
I can soothe my baby when he/she is distressed	☐ 0	☐ 1	☐ 2	☐ 3	☐ 4
I am confident about playing with my baby	☐ 0	☐ 1	☐ 2	☐ 3	☐ 4
If my baby has a cold or slight fever, I am confident about handling this	☐ 0	☐ 1	☐ 2	☐ 3	☐ 4
I feel sure that my partner will be there for me when I need support	☐ 0	☐ 1	☐ 2	☐ 3	☐ 4
I am confident that my baby is doing well	☐ 0	☐ 1	☐ 2	☐ 3	☐ 4
I can make decisions about the care of my baby	☐ 0	☐ 1	☐ 2	☐ 3	☐ 4
Being a mother is very stressful for me	☐ 0	☐ 1	☐ 2	☐ 3	☐ 4
I feel I am doing a good job as a mother	☐ 0	☐ 1	☐ 2	☐ 3	☐ 4
Other people think I am doing a good job as a mother	☐ 0	☐ 1	☐ 2	☐ 3	☐ 4
I feel sure that people will be there for me when I need support	☐ 0	☐ 1	☐ 2	☐ 3	☐ 4

The THRIVE Trial is funded by the National Institute for Health Research's Public Health Research Programme

89 **Below is a series of statements about being a mother. In each case, please tick the answer which most applies to you.**
(Please tick **one box per line**)

	Strongly agree	Agree	Disagree	Strongly disagree
I think my baby is very demanding	1	2	3	4
I feel proud of being a mother	1	2	3	4
I am disappointed by motherhood	1	2	3	4
Having a baby has made me as happy as I expected	1	2	3	4
I sometimes regret having my baby	1	2	3	4
I am the only person who can look after my baby properly	1	2	3	4
To be a good mother, I should be able to cope well all the time	1	2	3	4
If my baby is unwell or unhappy it is not my fault	1	2	3	4
I have resented not having enough time to myself since having my baby	1	2	3	4
My daily life has been no more difficult since my baby was born	1	2	3	4
If I find being a mother difficult, I feel a failure	1	2	3	4
If I love my baby I should want to be with him/her all the time	1	2	3	4
If other people help me look after my baby, I feel a failure	1	2	3	4
I resent the way my life has been restricted since having my baby	1	2	3	4

The THRIVE Trial is funded by the National Institute for Health Research's Public Health Research Programme

90 **The following questions are about things that sometimes cause problems for parents in the first few months after their baby is born. Thinking back, how well do you think you coped with the problems listed in the first three months after your baby was born?**

(Please tick **one box per line**)

	Very well	Quite well	Not well	Not well at all	Does not apply
managing the relationship between my baby and his/her brothers and sisters	1	2	3	4	5
my baby suffering from wind or colic	1	2	3	4	5
my baby's sleeping pattern	1	2	3	4	5
getting my baby to feed	1	2	3	4	5
my baby having health problems	1	2	3	4	5
being able to afford all the clothes and equipment I needed for my baby	1	2	3	4	5
managing the house and other domestic responsibilities (eg cooking, cleaning, shopping)	1	2	3	4	5
problems with teething	1	2	3	4	5

91 **Which best describes your feelings during the first few weeks after your baby was born?**

(Please tick **one box only**)

- My baby still seemed a part of me ☐ 0
- My baby seemed an outgoing, sociable person ☐ 1
- My baby seemed separate but not yet sociable ☐ 2

The THRIVE Trial is funded by the National Institute for Health Research's Public Health Research Programme

92	**Most people find being a parent has its ups and downs. Taking everything into account, which of the statements best describes how you are coping with being a parent these days?** (Please tick **one box only**)		
	I feel I am not coping at all these days	☐	1
	Most of the time I feel I am not coping very well	☐	2
	Sometimes I feel I am coping but sometimes things get on top of me	☐	3
	Most of the time I feel I am coping pretty well	☐	4
	I always feel I am coping really well – things never or hardly ever get on top of me	☐	5
	Don't know	☐	6

93	**We are interested in how parents think and feel when their babies cry. Please tick the box that best describes how often you tend to think or feel this way when your baby cries.**

When my baby cries...

(Please tick **one box per line**)

	Never	Rarely	Sometimes	Often	Always
I want my baby to know he/she can rely on me to help	1	2	3	4	5
I want to make my baby stop quickly because crying is a nuisance	1	2	3	4	5
I want to make my baby feel secure/cared for	1	2	3	4	5
I want to make my baby stop so others aren't disturbed	1	2	3	4	5
I let my baby cry it out so he/she doesn't get too dependent on me	1	2	3	4	5
I want to make my baby feel better because it makes me feel like a good parent	1	2	3	4	5
I will just remind myself babies don't have feelings	1	2	3	4	5
I want my baby to stop crying because I am not sure I know the right way to respond	1	2	3	4	5
I think my baby is trying to tell me something	1	2	3	4	5

The THRIVE Trial is funded by the National Institute for Health Research's Public Health Research Programme

When my baby cries...
(Please tick **one box per line**)

	Never	Rarely	Sometimes	Often	Always
I know it's for a physical reason like needing to be fed, changed, or take a nap and not for an emotional reason like feeling sad or afraid	1	2	3	4	5
I want to make my baby stop crying because it shows people I'm a good parent	1	2	3	4	5
I think my baby just wants attention	1	2	3	4	5
I think my baby is trying to communicate with me	1	2	3	4	5
I think my baby is trying to control or manipulate me	1	2	3	4	5
I want to make my baby feel better	1	2	3	4	5
I want my baby to stop because I can't get anything else done	1	2	3	4	5
I want to make my baby feel safe	1	2	3	4	5
I want my baby to stop because crying doesn't accomplish anything	1	2	3	4	5
I want to comfort my baby	1	2	3	4	5
I think my baby is crying for a reason	1	2	3	4	5
I let my baby cry it out so he/she doesn't get spoiled	1	2	3	4	5

The THRIVE Trial is funded by the National Institute for Health Research's Public Health Research Programme

94 **We also want to know why parents decide how to respond when their babies cry. Please tick the box that best describes how often you have felt or thought the following things when you respond to your baby's cries.**

The way I respond when my baby cries...

(Please tick **one box per line**)

	Never	Rarely	Sometimes	Often	Always
can spoil my baby	1	2	3	4	5
can affect how my baby feels about him/herself in the future	1	2	3	4	5
teaches my baby about emotions (like how to show them appropriately)	1	2	3	4	5
lets my baby know that I am in charge	1	2	3	4	5
helps my baby learn how to cope with emotions	1	2	3	4	5
shows what a good parent I am	1	2	3	4	5
makes my baby feel safe and secure	1	2	3	4	5
can affect how my baby feels about me in the future	1	2	3	4	5
lets my baby know that it is okay to be upset	1	2	3	4	5
lets my baby know that there is no good reason to cry	1	2	3	4	5

The THRIVE Trial is funded by the National Institute for Health Research's Public Health Research Programme

**The way I respond when my baby cries...** (Please tick **one box per line**)					
	**Never**	**Rarely**	**Sometimes**	**Often**	**Always**
makes my baby feel like he/she can rely on me	1	2	3	4	5
helps me get on with other things	1	2	3	4	5
helps my baby move on to more important things like learning and exploring	1	2	3	4	5
is more important to me than my baby	1	2	3	4	5
makes my baby feel like I care about how he/she feels	1	2	3	4	5
teaches my baby that it is just not okay to throw a fit	1	2	3	4	5
teaches my baby to control his/her emotions	1	2	3	4	5
makes my baby feel confident	1	2	3	4	5
helps my baby move on to having fun	1	2	3	4	5
teaches my baby that crying doesn't get you what you want	1	2	3	4	5
teaches my baby how to get along with other people	1	2	3	4	5
has no long term effect on my baby	1	2	3	4	5

The THRIVE Trial is funded by the National Institute for Health Research's Public Health Research Programme

95 **How would you describe your interaction with your baby?**
(Please tick **one box only**)

I adapt myself to my baby	☐	0
We (my baby and me) negotiate between us	☐	1
The baby adapts to the household routine	☐	2

96 **The following questions are about your relationship with your baby. Thinking about your relationship with your baby now, please read each statement carefully and tell us how often the statement is true.**
(Please tick **one box per line**)

	Always	Very often	Quite often	Some times	Rarely	Never
I feel close to my baby	☐ 1	☐ 2	☐ 3	☐ 4	☐ 5	☐ 6
I wish the old days when I had no baby would come back	☐ 1	☐ 2	☐ 3	☐ 4	☐ 5	☐ 6
I feel distant from my baby	☐ 1	☐ 2	☐ 3	☐ 4	☐ 5	☐ 6
I love to cuddle my baby	☐ 1	☐ 2	☐ 3	☐ 4	☐ 5	☐ 6
My baby winds me up	☐ 1	☐ 2	☐ 3	☐ 4	☐ 5	☐ 6

The THRIVE Trial is funded by the National Institute for Health Research's Public Health Research Programme

	Always	Very often	Quite often	Some times	Rarely	Never
My baby irritates me	1	2	3	4	5	6
I feel happy when my baby smiles or laughs	1	2	3	4	5	6
I love my baby to bits	1	2	3	4	5	6
I enjoy playing with my baby	1	2	3	4	5	6
I feel trapped as a mother	1	2	3	4	5	6
I feel angry with my baby	1	2	3	4	5	6
I resent my baby	1	2	3	4	5	6
My baby is the most beautiful baby in the world	1	2	3	4	5	6
My baby makes me anxious	1	2	3	4	5	6
My baby annoys me	1	2	3	4	5	6
My baby is easily comforted	1	2	3	4	5	6

The THRIVE Trial is funded by the National Institute for Health Research's Public Health Research Programme

97 **The next few questions ask for your views on bringing up young children. Please say how much you agree or disagree with each one.**

(Please tick **one box per line**)

	Strongly agree	Agree	Neither agree nor disagree	Disagree	Strongly disagree
Nobody can teach you how to be a good parent, you just have to learn for yourself	0	1	2	3	4
It's more important to go with what the child wants than to stick to a firm routine for feeding and sleeping	0	1	2	3	4
It's better for children to have two parents than one	0	1	2	3	4
If you ask for help or advice on parenting from professionals like doctors or social workers they start interfering or trying to take over	0	1	2	3	4
It's difficult to ask people for help or advice about parenting unless you know them really well	0	1	2	3	4
It's hard to know who to ask for help or advice about being a parent	0	1	2	3	4
It may not be a good thing to smack but sometimes it's the only thing that will work	0	1	2	3	4

The THRIVE Trial is funded by the National Institute for Health Research's Public Health Research Programme

98 **Does your baby have a daily routine?**

(Please tick **one box only**)

Yes ☐ 1 **Go to Question 99**

No ☐ 2 **Go to Question 101**

99 **How old was your baby when this routine began?**

(Please **write in your answer**, if you can't remember write in your 'best guess')

__________ months __________ weeks __________ days old

100 **Please describe your baby's usual daily routine.**

(Please **write in**)

The THRIVE Trial is funded by the National Institute for Health Research's Public Health Research Programme

You and your household

101 **How many times have you moved house in the last year?**
(Please tick **one box only**)

I have not moved house	☐	1	**Go to Question 104**
Once	☐	2	**Go to Question 102**
Twice	☐	3	**Go to Question 102**
Three times	☐	4	**Go to Question 102**
More than three times	☐	5	**Go to Question 102**
I can't remember if I have moved house	☐	6	**Go to Question 102**

102 **I am currently living...**
(Please tick **all that apply**)

in a house or flat that is owned outright	☐	1
in a house or flat that is being bought with the help of a mortgage or loan	☐	1
in a house or flat rented from a council, local authority or housing association	☐	1
in a house or flat rented from a private landlord	☐	1
at home with my parents	☐	1
rent free with a family member or friend	☐	1
in a hostel, bed and breakfast, homeless shelter or temporary accommodation	☐	1
in a children's unit, foster care placement or supported care placement	☐	1
other (please **write in**)	☐	1

____

The THRIVE Trial is funded by the National Institute for Health Research's Public Health Research Programme

103	**How many rooms are there in your home, excluding the kitchen and bathroom?** (Please **write in**)

______________ rooms

104	**How much of a problem do you have with damp, mould or condensation on the walls in your home, apart from in the kitchen or bathroom?** (Please tick **one box only**)

None, there is no damp ☐ 1

Not much of a problem ☐ 2

Some problem ☐ 3

Great problem ☐ 4

105	**Which of these statements best describes you?** (Please tick **one box only**)

I am currently in paid employment or self-employed ☐ 1

I am currently on maternity leave from paid employment and plan to return to work ☐ 2

I am currently on maternity leave from paid employment but I do not plan to return to work ☐ 3

I am not currently working but have been in paid employment in the past ☐ 4

I have never been in paid employment ☐ 5

The THRIVE Trial is funded by the National Institute for Health Research's Public Health Research Programme

106	How many people in total (including yourself and all children of all ages) live here most of the time as members of this household?

______________ Persons

For each member of the household, excluding yourself and your 6 month old baby, could you tell me:

	**Their relationship to you** e.g. partner, daughter, son or friend	**Their relationship to your baby** e.g. father, sister or grandparent	**Sex** M	**Sex** F	**Age**	**What do they do?** (Please tick **one box only**) Pre-school	School	College/ university	At work /training	Unemployed	Retired	House-person	Other
Example 1	**Partner**	**Dad**	✓ 1	2	**28**	1	2	3	4	✓ 5	6	7	8
Example 2	**Daughter**	**Sister**	1	✓ 2	**6**	1	✓ 2	3	4	5	6	7	8
			1	2		1	2	3	4	5	6	7	8
			1	2		1	2	3	4	5	6	7	8
			1	2		1	2	3	4	5	6	7	8
			1	2		1	2	3	4	5	6	7	8
			1	2		1	2	3	4	5	6	7	8
			1	2		1	2	3	4	5	6	7	8
			1	2		1	2	3	4	5	6	7	8
			1	2		1	2	3	4	5	6	7	8
			1	2		1	2	3	4	5	6	7	8

The THRIVE Trial is funded by the National Institute for Health Research's Public Health Research Programme

Taking part in the THRIVE trial

107 **Think back to when you first heard about the study and the thoughts and feelings that you had at that time. It would help us if you answered all items as best you can even if you are not absolutely certain.**
(Please tick **one box per line**)

	Not at all	A little bit	Moderately	Quite a bit	Extremely
I was happy that I was asked to take part in the study	1	2	3	4	5
I didn't really want to take part but I felt I had to.	1	2	3	4	5
I was happy when I found out which group I would be in.	1	2	3	4	5

The THRIVE Trial is funded by the National Institute for Health Research's Public Health Research Programme

Please detach this page and give it to the researcher who will file it separately from the rest of the questionnaire

What is your date of birth?
(Please **write in** – for example: 30/03/1983)

☐ ☐ / ☐ ☐ / ☐ ☐ ☐ ☐

Do you know your postcode? If so, tick 'yes' and write it down, if no tick 'no'. If you only know the beginning then please write this in.

☐ 1 **Yes**, my postcode is: ☐ ☐ ☐ ☐ ☐ ☐ ☐

e.g.	K	A	I	4	8	R	J
		G	I	2	8	R	Z

☐ 2 **No**

The THRIVE Trial is funded by the National Institute for Health Research's Public Health Research Programme

END OF QUESTIONNAIRE

Thank you very much for taking part and answering the questions

If you have any thoughts on this questionnaire or feedback for the THRIVE team that may help us inform future aspects of this research, please feel free to leave comments here. You can also email your comments to THRIVE@sphsu.mrc.ac.uk

Your use of services

How many times have you, or your child, visited the following services, since the last time we saw you?
If you cannot remember the exact number of visits, don't worry, please just give your best guess.

Number of visits/appointments or calls for	You	Child
**Example:** GP	1	2
**HEALTH - NHS**		
Family doctor (GP)		
Dentist visits		
Health visitor (clinic visit)		
Health visitor (home visit)		
Health visitor (phone call)		
Midwife (phone call)		
Midwife (home visit)		
Midwife (clinic/hospital visit)		
Hospital (total number of nights spent in hospital)		
Hospital (day visit)		
Hospital (A&E visit)		
GP out of hours/minor injury unit		
NHS 24 phone calls		
Pharmacy visits (for prescription medication)		
**HEALTH - SPECIALITY NHS**		
Physiotherapist		
Paediatrician (children's doctor)		
Obstetrician (pregnancy doctor)		
Dietician (food & nutrition specialist)		
Diabetic specialist (nurse or doctor)		
Speech and language specialist		
Hearing specialist		
Eye specialist		
NHS smoking cessation		
NHS antenatal classes		
Private antenatal classes (e.g. NTC, Lazy Daisy)		

Number of visits/appointments or calls for	You	Child
**MENTAL HEALTH SERVICES**		
Clinical psychologist		
Psychiatrist		
Community Psychiatric Nurse		
Perinatal mental health team		
SAMH (Scottish Association for Mental Health)		
Mother and Child Unit		
CAMHS (Child Adolescent Mental Health Services)		
Learning Disability CAMHS		
Bereavement Counselling/Support		
Stress Management (e.g. Life Links)		
Counsellor/Therapist		
**SOCIAL SUPPORT/JUSTICESERVICES**		
Social worker (home visit)		
Social worker (at a social work office)		
Social worker (phone call)		
Community addictions team		
Alcohol/drug support (e.g. Addaction)		
Pre-birth case conference		
Children's panel/case conference (in court)		
Children's panel/case conference (not in court)		
Homestart		
Women's Aid		
Women's protection services (e.g. a refuge)		
ASSIST		
Police call outs		
Court attendances		

Number of visits/appointments or calls for	You	Child
**ANTENATAL/PARENTING SUPPORT**		
Young parent support groups		
Barnardo's Threads		
Family support groups (e.g. Quarriers)		
Family Nurse Practitioner		
Minding the Child		
Mellow Bumps, not run by the THRIVE trial		
Mellow Babies, Mellow Mums or Mellow Toddlers		
Triple P for Child, not run by the THRIVE trial		
A Triple P seminar or group (not Triple P for Child)		
Mother & child group		
Child Massage		
Rhyme time/ nursery rhyme classes		
**CONSUMER SERVICES**		
Housing Association (phone call)		
Housing Association (at housing office)		
Housing Association (home visit)		
Citizens Advice Bureau		
Legal Aid		
**LIFESTYLE**		
Cookery classes		
Exercise classes		
Budgeting/ managing money classes		
Adult education classes		
**PRE-SCHOOL / CHILD CARE**		
State nursery (free/ reduced cost)		
Private nursery (paid)		
Teacher-counsellor (pre-school)		

PLEASE COMPLETE FOSTER CARE HISTORY FOR CHILD

Has your child been in foster care? YES NO
(please circle)

Number of foster care placements ________________
(please write number)

PLACEMENT 1
Age when placed into foster care ____________ months
Age when left foster care ____________ months

PLACEMENT 2
Age when placed into foster care ____________ months
Age when left foster care ____________ months

PLACEMENT 3
Age when placed into foster care ____________ months
Age when left foster care ____________ months

How much do you spend per month on over the counter medication?

For you £ ____________

For child £ ____________

Please record details of any services used but not listed

The THRIVE Trial is funded by the National Institute for Health Research's Public Health Research Programme

The THRIVE Trial is funded by the National Institute for Health Research's Public Health Research Programme
